# The *Pratylenchus penetrans* Transcriptome as a Source for the Development of Alternative Control Strategies: Mining for Putative Genes Involved in Parasitism and Evaluation of *in planta* RNAi

**DOI:** 10.1371/journal.pone.0144674

**Published:** 2015-12-14

**Authors:** Paulo Vieira, Sebastian Eves-van den Akker, Ruchi Verma, Sarah Wantoch, Jonathan D. Eisenback, Kathryn Kamo

**Affiliations:** 1 Dept. of Plant Pathology, Physiology, and Weed Science, Virginia Tech, Blacksburg, VA, 24061, United States of America; 2 Floral and Nursery Plants Research Unit, U.S. National Arboretum, U.S. Department of Agriculture, Beltsville, MD, 20705–2350, United States of America; 3 School of Life Sciences, University of Dundee, Dundee, DD1 5EH, United Kingdom; James Hutton Institute, UNITED KINGDOM

## Abstract

The root lesion nematode *Pratylenchus penetrans* is considered one of the most economically important species within the genus. Host range studies have shown that nearly 400 plant species can be parasitized by this species. To obtain insight into the transcriptome of this migratory plant-parasitic nematode, we used Illumina mRNA sequencing analysis of a mixed population, as well as nematode reads detected in infected soybean roots 3 and 7 days after nematode infection. Over 140 million paired end reads were obtained for this species, and *de novo* assembly resulted in a total of 23,715 transcripts. Homology searches showed significant hit matches to 58% of the total number of transcripts using different protein and EST databases. In general, the transcriptome of *P*. *penetrans* follows common features reported for other root lesion nematode species. We also explored the efficacy of RNAi, delivered from the host, as a strategy to control *P*. *penetrans*, by targeted knock-down of selected nematode genes. Different comparisons were performed to identify putative nematode genes with a role in parasitism, resulting in the identification of transcripts with similarities to other nematode parasitism genes. Focusing on the predicted nematode secreted proteins found in this transcriptome, we observed specific members to be up-regulated at the early time points of infection. In the present study, we observed an enrichment of predicted secreted proteins along the early time points of parasitism by this species, with a significant number being pioneer candidate genes. A representative set of genes examined using RT-PCR confirms their expression during the host infection. The expression patterns of the different candidate genes raise the possibility that they might be involved in critical steps of *P*. *penetrans* parasitism. This analysis sheds light on the transcriptional changes that accompany plant infection by *P*. *penetrans*, and will aid in identifying potential gene targets for selection and use to design effective control strategies against root lesion nematodes.

## Introduction

Worldwide crop losses due to plant-parasitic nematodes have been estimated at $118 billion annually, with root lesion nematodes (RLN), *Pratylenchus* spp., ranking third in terms of economic losses. The root lesion nematode *P*. *penetrans* (Cobb, 1917) Filipjev and Shuurmans Stekhoven, 1941 is considered one of the most economically important species within the genus. Host range studies have shown that nearly 400 plant species can be parasitized by *P*. *penetrans* [1]. This species presents a wide geographic distribution, and is often reported as a limiting factor for the production of several important agronomic [e.g. alfalfa (*Medicago sativa* L.), bean (*Phaseolus vulgaris* L.), corn (*Zea mays* L.), potato (*Solanum tuberosum* L.)] or ornamental crops [e.g. lily (*Lilium candidum* L.), boxwood (*Buxus sempervirens* L.)] and fruit trees [e.g. apple (*Malus domestica* Borkh.), peach (*Prunus persica* (L.) Batsch.)] [1]. In the USA *P*. *penetrans* is considered one of the most important plant-parasitic nematodes in the Pacific Northwest affecting the production of a range of crops (e.g. potato, raspberries (*Rubus idaeis* L.), lilies). In Europe this species has been recently detected in several potato fields in Portugal, with the total number of nematodes found in the soil at or above threshold levels considered to be a potential treat to crop production [2]. In agreement with the Food and Environment Research Agency recent reports, this species has been also linked to scab in UK, impacting the marketable quality of potatoes [3].

*Pratylenchus* spp. are migratory endoparasitic nematodes that feed and migrate within the root cortical tissue causing a reduction in root growth after infection, accompanied by the formation of lesions, necrotic areas, browning and cell death [4]. As migratory endoparasites these nematodes destroy tissues of the root system causing surface openings that allow secondary attack by soil pathogens, such as fungi [5] or bacteria [6]. Like other nematodes, the life cycle of *P*. *penetrans* is punctuated by six stages (eggs, four juvenile stages and adults). Although the majority of *Pratylenchus* species reproduce by parthenogenesis, *P*. *penetrans* reproduces sexually [1]. With the exception of eggs and J1 stages, all the remaining juvenile and adult stages are vermiform and motile, allowing them to infect host plants [1].

Currently, the most common strategies used for RLN control are genetic resistance, nematicide application, and rotation with non-host crops [7]. Host resistance to *Pratylenchus* spp. is very limited, as only a few *loci* have been linked to resistance/tolerance to some RLN species, such as in wheat (*Triticum aestivum* L.) [8] or barley (*Hordeum vulgare* L.) [9]. Application of chemicals to control RLN is not a sustainable option, as most of these chemicals increase production costs and present negative effects to the environment.

With the increased knowledge from data generated by next-generation sequencing technology (454 and Illumina), the comparison between the molecular actors within plant-parasitic nematode species will bring new avenues for a better understanding of their relationship with the host and establishment of their associated diseases. Although a greater number of studies have been devoted to sedentary plant-parasitic nematode species using such methodologies, transcriptome analyses have been conducted for migratory nematode species, including *Pratylenchus coffeae* Goodey, 1951 [10], *P*. *thornei* Sher and Allen, 1953 [11], and *P*. *zeae* Graham, 1951 [12]. More recently, the genome of *P*. *coffeae* has been released [13], revealing a reduced genome of 19.67 Mb [14], encoding for approximately 6712 genes [13]. So far for *P*. *penetrans* only a small EST dataset from a mixed-stage population containing 1928 contigs has been generated and published for this species [15].

The parasitism strategy of *Pratylenchus* spp. suggests a less specialized nematode-host interaction, possibly representing an evolutionary intermediate step between the highly specialized sedentary plant-parasitic and the free-living nematodes [1]. Although sedentary and migratory plant-parasitic nematodes share common elements, the migratory nematodes do not induce specialized nematode feeding sites (e.g. giant cells or syncytia). Invasion of plant tissue by *Pratylenchus* spp. is generally thought to involve both mechanical force from the robust stylet and secretion of a repertoire of nematode proteins through the stylet. Consistent with other plant-parasitic nematodes, root lesion nematodes produce an array of cell wall modifying enzymes (CWMEs), which are believed to facilitate host cell wall degradation and migration of nematodes along the root tissues [10,11,12]. However, the molecular mechanisms of pathogenicity of root lesion nematodes are still poorly understood, and this may be attributed to the limited knowledge and functional analyses of their genes.

Herein we report overall analyses of the transcriptome of *P*. *penetrans* generated by pair-end Illumina sequencing and *de novo* assembly, followed by annotation and comparative analyses to other nematode species. We explore the efficacy of RNAi, delivered from the host, as a strategy to control the migratory nematode *P*. *penetrans*, by targeted knock-down of selected nematode genes. We annotate putative “parasitism genes” by sequence homology to those of related species. Finally, we conduct a *de novo* identification of putative parasitism genes based on differential expression and specific up-regulation during the early phases of plant infection.

## Materials and Methods

### Nematode collection and RNA extraction processed for Illumina library preparation and sequencing

Mixed populations (eggs, juveniles and adults) of *P*. *penetrans* were extracted from roots of soybean plants (*Glycine max* (L.) Merr. “Williams 82”) grown in sterile conditions using a modified tray method [16], with nematodes collected over the course of three to five days. *Pratylenchus penetrans* isolate NL 10p RH was originally collected from soil samples located in Beltsville (Maryland, US), identified to species level and provided by the Nematology Laboratory (USDA-ARS, USA). For mRNA-seq analysis two nematode samples were processed as biological replicates for RNA extraction and further sequencing. Nematode RNA extraction was carried out using RNeasy Spin columns (Qiagen, Valencia, CA, USA) following the manufacturer’s instructions for animal tissues using the optional on-column DNase I digestion (Qiagen, Valencia, CA, USA). In a parallel study (Vieira et al. unpublished), RNA was extracted from whole soybean root systems, infected with 400 nematodes, 3 and 7 days after infection (DAI), following the same protocol, each in biological duplicate. RNA quality of all samples was assessed using a Nanodrop spectrophotometer (NanoDrop) and a Bioanalyser (Agilent Technologies). RNA samples showing absorbance ratios of 260:280 nm and 260:230 nm above 2, and an integrity value (RIN) above 9 were used for sequencing using the service provided by LCSciences (Houston, Texas, USA). Sequencing libraries were generated using 2 μg of total RNA (per sample) following the manufacturer’s instructions (Illumina Inc., San Diego, CA) and 100 base pair paired-ends were sequenced on an Illumina platform. Raw sequencing reads have been deposited at the National Center for Biotechnology Information NCBI under BioProject ID PRJNA304159.

### *De novo* transcriptome assembly and analysis

Reads derived from the two nematode samples were assessed for quality, with adapter sequences, low quality sequences with ambiguous bases, and shorter than 50 bp being excluded. Using Trinity, reads from nematode samples were assembled using a minimum Kmer coverage of 25 to produce a collection of contigs representing the pool of cDNA fragments generated in this study [17]. The quality of the assembly was assessed by LC Sciences before subsequent analysis, and overall redundancy was reduced by removal of transcripts wholly represented in other transcripts at 98% similarity (CD HIT-EST) [18]. Only transcripts with sequence length longer than 200 bp were then considered for annotation. Trimmed RNA reads (Phred > 22) from each nematode sample were independently mapped back to the non-redundant assembly using Bowtie2. For *in planta* samples, only those reads that did not map to the *Glycine max* Williams 82 reference genome (Wm82.a1), download from the portal http://www.phytozome.net/soybean, were used for further analyses. The abundance of assembled transcripts in each condition was estimated using Trinity wrapper scripts for RSEM (RNA-seq by expectation-maximization) [19]. Transcript expression was TMM (trimmed mean of *M*-values) normalized, and those significantly differentially expressed between conditions with a minimum fold change of 2 were identified using EdgeR (False Discovery Rate (FDR) P<0.001). Significantly differentially expressed transcripts were grouped into clusters based on 15 percent of tree height using accompanying Trinity scripts.

### Functional annotation of transcripts

The full set of obtained transcripts was annotated using Blastx (E-value < 1e^-5^ and bitscore > 50) against the NCBI non-redundant protein (NR) (http://www.ncbi.nlm.nih.gov/) [20] and Swiss-Prot (http://www.uniprot.org/) [21] databases. Local tBlastx analyses (E-value < 1e^-5^ and bitscore > 50) were performed against all Expressed Sequence Tags (EST) database for Nematoda downloaded from NCBI or from the NEMBASE4 database (http://www.nematodes.org/nembase4/), a nematode transcriptome database derived from whole genome sequencing and EST assemblies [22] in order to identify similarities to the other species of the Phylum Nematoda.

*Pratylenchus penetrans* transcripts were also subject to similarity search analyses against predicted protein datasets of complete nematode annotated genomes. Databases used for sequence comparison included plant-parasitic nematodes: *Bursaphelenchus xylophilus* (Steiner and Burher, 1934) Nickle, 1971 [23], *Globodera pallida* (Wollenweber, 1923) Stone, 1973 [24], *Meloidogyne incognita* (Kofoid and White, 1919) Chitwood, 1949 [25], *Meloidogyne hapla* Chitwood, 1949 [26]; an animal-parasitic nematode: *Brugia malayi* S.L. Brug, 1927 [27]; and the free-living nematode, *Caenorhabditis elegans* (Maupaus, 1900) Dougherty, 1955 [28]. Transcriptome data generated for other *Pratylenchus* species; in particular *Pratylenchus coffeae* (25,987 contigs) [10], *P*. *thornei* (6,667 contigs) [11], and *P*. *zeae* (10,163 contigs and 139,104 singletons) [12] were also included for pairwise tBlastx analyses. In the case of *P*. *vulnus* sequences were obtained directly from NCBI EST database (5,630 sequences). The *E*-value cutoff of 1e^-5^ and bitscore > 50 was used to accept sequence similarities in all BLAST searches.

### Functional classification of transcripts using Gene Ontology and Ortholog Comparison

Following the tBlastx searches, the set of transcripts were assigned to hierarchical gene ontology (GO) terms on the basis of biological processes, molecular functions, and cellular components. Functional annotation by GO terms was analyzed by BLAST2GO software, using default parameters [29]. InterPro Scan was also performed to match *P*. *penetrans* transcripts to characterized protein domains in the InterPro database using BLAST2GO software.

### *Pratylenchus penetrans* transcriptome data mining

Nematode secreted proteins with a putative role in parasitism were identified based on the above Blast comparisons against other plant-parasitic nematode species or by using individual sequences of particular known nematode candidates [30]. Both best-hit and individual nematode sequences were manually inspected. In addition, all transcripts were translated using the OrfPredictor tool [31] with the longest open reading frame (ORF) extracted. In order to identify further putative nematode secreted proteins, the presence of signal peptides was predicted with SignalP 4.0 [32] and transmembrane domains were predicted using TMHMM server version 2.0. The CAZymes Analysis Toolkit [33] was used to detect transcripts encoding other putative carbohydrate active enzymes (E-value threshold of E^-5^ and a bitscore threshold of 55). The MEROPS database was used to identify putative *P*. *penetrans* peptidases [34], using an E-value < 1e^-5^ for nematode transcripts containing a signal peptide and no TMHMM. For the identification of *C*. *elegans* neuropeptide putative homologues belonging to the FMRFamide-like, NLP-like and insulin-like peptides families [35], as well as putative homologues of the *C*. *elegans* RNAi pathway [36], protein sequences were downloaded from Wormbase (release WS248, www.wormbase.org) and used as queries against the *P*. *penetrans* transcriptome.

### Semi-quantitative RT-PCR validation of a selected set of predicted *Pratylenchus penetrans* genes

Primers complementary to a predicted set of transcripts were designed based on the assembled *P*. *penetrans* transcriptome and used to amplify fragments of 11 predicted genes (S1 Table). Total RNA was extracted from a mixed population of *P*. *penetrans*, soybean infected roots, or from separated developmental stages (eggs, juveniles, females and males) using the RNeasy Plant Mini Kit (Qiagen, Valencia, CA, USA) following the manufacturer’s instruction. RNA was then treated with RNAase-Free DNase (Qiagen, Valencia, CA, USA) before reverse transcription. RNA was added to the RT reaction using the iScript first-strand synthesis kit (Bio-Rad, Hercules, CA, USA) to produce cDNA, which was posteriorly used for semi-quantitative RT-PCR analyses. *P*. *penetrans 18S* gene was used as reference gene.

### *In planta* nematode RNAi gene silencing assays

An RNAi-mediated nematode-silencing assay was performed for two locomotion-related genes of *P*. *penetrans* (*Pp-pat-10* and *Pp-unc-87*), previously targeted for other *Pratylenchus* species using a nematode soaking methodology [37,38]. The templates for the production of the dsRNA constructs were amplified using the cDNA samples previously generated. The nematode gene fragments were individually cloned into the pENTR vector (Invitrogen). Clones were checked through DNA sequencing and transferred to the pRAP17 vector [39]. The cloning reaction was mediated using the Gateway^®^ LR Clonase^™^ Enzyme Mix (Invitrogen, Carlsbad, CA, USA). Transformation of each individual nematode fragment into the pRAP17 vector was confirmed by PCR using the relevant primer pairs (S1 Table). The pRAP17 constructs were then individually transferred to competent *Agrobacterium rhizogenes* (K559) and transformations were confirmed by PCR using the same set of primers (data not shown). Soybean hairy root lines were then generated for each dsRNA nematode construct, while control hairy root lines were generated using *A*. *rhizogenes* harbouring an empty vector (pBIN-JIT) with kanamycin resistance to both bacteria and plants [40]. Generation of soybean hairy roots was carried out according to [41]. Transformed hairy roots expressing the dsRNA constructs were initially identified by eGFP fluorescence, while control hairy roots where selected based on kanamycin (200 μg/ml) growing in Murashige and Skoog (MS) selective medium plates.

For confirmation of the nematode gene fragment in the transformed hairy roots, genomic DNA was isolated for PCR amplification using the FastDNA kit (MP Biomedicals). Afterwards, total RNA was isolated from 100 mg of fresh soybean hairy roots using the RNeasy Plant Mini Kit (Qiagen, Valencia, CA, USA) following the manufacturer’s instructions. The RNA was treated with RNase-Free DNase (Qiagen, Valencia, CA, USA) before reverse transcription. One microgram of treated RNA was added to the RT reaction using the First-Strand cDNA Synthesis kit (Invitrogen, USA) as described by the manufacturer. The oligonucleotide primers specific for the intron of the pRAP17 vector was used to synthesize the first cDNA strand for each transformed soybean hairy root line, and the corresponding cDNAs were used as a template for amplification of a 241 bp fragment using the same primers. After confirming the presence of the transgene by PCR and expression of the intron that separates each fragment by semi-quantitative RT-PCR, three independent RNAi lines were selected for nematode resistance assays.

### Nematode RNAi gene silencing assays

For nematode resistance tests, roots 3–5 cm in length were excised from stock cultures and transferred to fresh MS plates without antibiotics. Three independent lines were challenged with RLN using nine hairy root systems per line in each of two independent biological tests for each dsRNA construct. Three control lines (containing the pBinJit empty vector) were selected using nine hairy roots per line. Two weeks later, each hairy root system was inoculated with a mixed population of approximately 300 sterile *P*. *penetrans* and maintained in the dark at 28°C. Approximately three months after nematode inoculation, infected soybean hairy roots were chopped into small pieces and both roots and media were placed into sterile glass bowls filled with sterile water containing 50 mg/l carbenicillin and 50 mg/l kanamycin. Nematodes were extracted 5 days later by sieving the water with a 500 μm mesh sieve. Data are expressed as the total mean number of nematodes ± Standard Error of the Mean (SEM) collected for each line, from two independent biological experiments. All data were analysed using analysis of variance (ANOVA), and means were compared using Tukey’s honestly significant difference (HSD) test at the 5% probability level.

For each nematode resistance test, a pool of the extracted nematodes growing in each corresponding soybean hairy root line were frozen immediately in liquid nitrogen and stored at -80°C. Total RNA was isolated and reverse transcribed to cDNA as described above. Transcript abundance of each nematode gene was then analyzed by qRT-PCR using two biological experiments, and three technical replicates were performed per assay. Real-time PCR reactions included 3.5 μL of SYBR green mix (Roche), 1 μL of 5 μM primers (S1 Table) and 100 ng of cDNA. Reactions were performed on a CFX96 Real-time system machine (BioRad). Expression levels were calculated using 2^-ΔΔCt^, and a student’s *t-*test was performed. *Pratylenchus penetrans’ 18S* ribosomal subunit was used for normalization of the qRT-PCR data.

## Results and Discussion

### 1. Transcriptome sequencing and *de novo* assembly

To obtain a global overview of the *P*. *penetrans* transcriptome, RNA collected from a mixed population (eggs, juveniles and adult stages) was prepared using two separated nematode samples, each sample containing thousands of individuals. Total RNA isolated from each nematode sample was used for sequencing using the Illumina platform, which resulted in a total of 149,688,264 raw reads (Table 1). The percentage of Q20 bases for the reads was >92%, with an average G + C content approximately of 43% (Table 1). After assembly, redundancy among transcripts was reduced using the CD-HIT-EST [18], and transcripts fully contained within other transcripts above 98% were discarded, resulting in a final set of 23,715 transcripts of ≥200 bp with a mean length of 882 bp and an N50 of 1,362 bp (Table 1).

**Table 1:** Overview of sequencing and *de novo* assembly results of mRNA-Seq analyses using RNA collected from *Pratylenchus penetrans*.

Sample	Total Bases	Read Count	GC (%)	Q20 (%)	Q30 (%)			
Pool of nematodes (sample 1)	7,916,194,968	78,378,168	43.21	92.18	87.37			
Pool of nematodes (sample 2)	7,202,319,696	71,310,096	43.84	93.56	87.65			
	**Trinity assembly K = 25**							
	**n**	**n: 100**	**n: N50**	**min**	**median**	**mean**	**N50**	**max**
***de novo* assembly**	**23,715**	**23,715**	**5,133**	**201**	**578**	**882**	**1,362**	**8,605**

Only sequences >200 bp were considered for this analysis.

### 2. Homology searches and individual nematode species comparative analyses

A Blastx search of the assembled transcriptome (n = 23,715) produced 12,361 (52.1%) transcripts with a significant Blastx hit to sequences deposited at the NR database, and 10,034 (42.3%) positive matches against Swissprot database (minimum *E*-value cutoff < 1e^-5^; and bitscore > 50). The relatively high percentage of transcripts with ‘no match’ obtained in our analyses is not surprising, as similar values have been reported for transcriptomes of other plant-parasitic species, including other root lesion nematode species [10,11,12]. Posteriorly, a tBlastx using the same set of transcripts was performed against the nematode EST database deposited at the NCBI containing 1,376,820 sequences, as well as to the annotated nematode sequences available in NEMBASE4 [22], with 237,181 sequences. In this case, a total of 13,964 (58%) and 13,260 (56%) transcripts showed significant hits to each respective database, increasing the final percentage of transcripts displaying a putative homologue within other nematode species.

The total set of *P*. *penetrans* transcripts was also compared with individual sets of proteins for a selected number of nematodes species with sequenced genome. A total of 45.8% of the sequences showed similarity (E-values < 1e^-5^ and bitscore above 50) to the protein sequences of the free-living nematode *C*. *elegans*, 44% to *B*. *malayi* (animal-parasitic nematode), 49.6% with *M*. *incognita* and 52% with *G*. *pallida* (sedentary plant-parasitic nematodes), and 48.6% with *B*. *xylophilus* (migratory plant-parasitic nematode).

**Fig 1:**
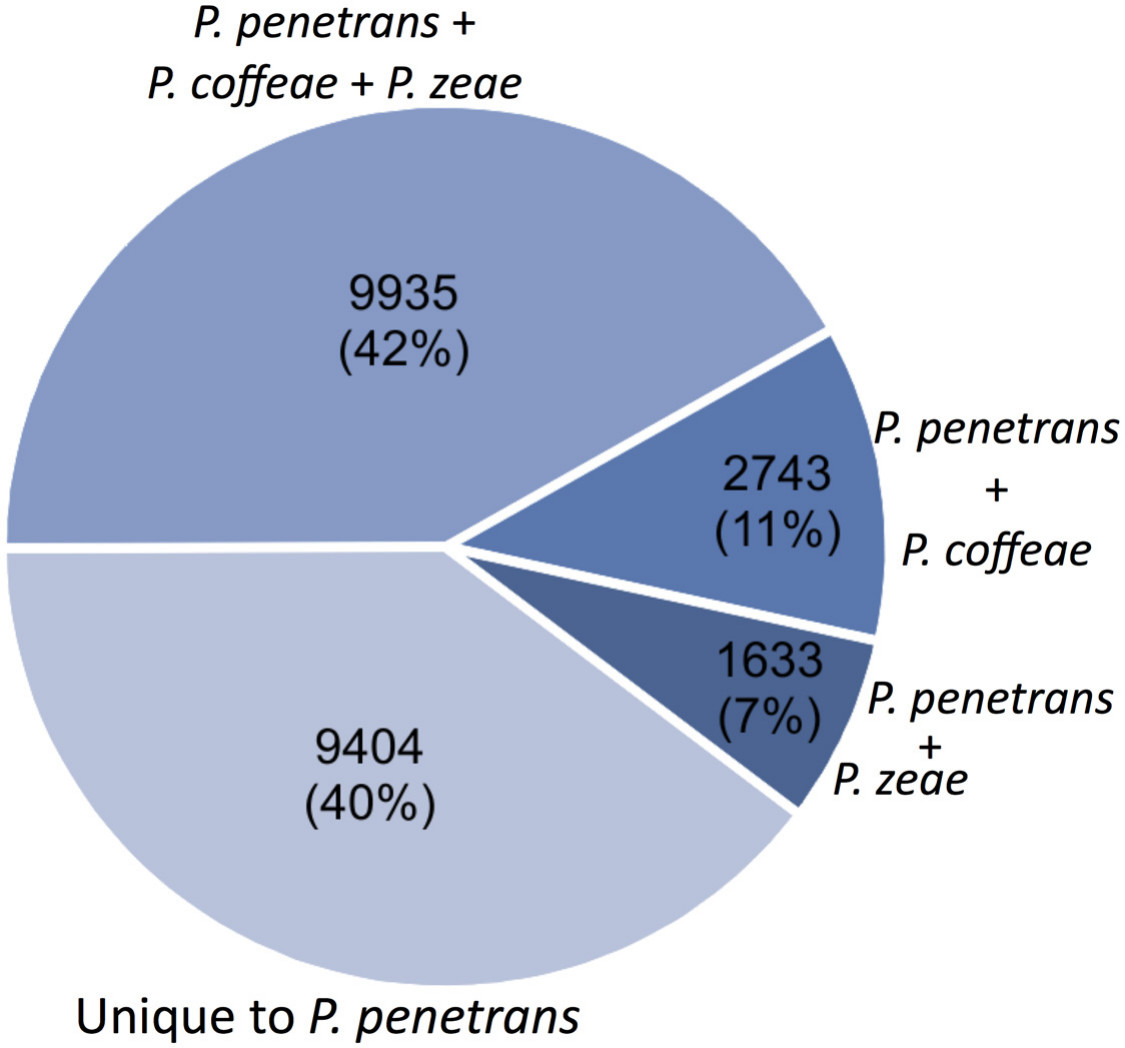
The number and percentage of *Pratylenchus penetrans* transcripts with similar sequences in related species. Using tBlastx (*E*-value cutoff < 1e^-5^; and bitscore > 50), 40% of the *P*. *penetrans* transcripts are not represented in either the transcriptome of *P*. *coffeae* [10] or that of *P*. *zeae* [13]. *P*. *penetrans* transcripts will similar sequences in both, or one of the related species is highlighted.

Within species of the genus *Pratylenchus*, a comparative analysis (Fig 1) was performed against the 454 *de novo* assemblies of the transcriptomes generated for *P*. *coffeae* [10] and *P*. *zeae* [12]. Out of the 23,715 transcripts generated for *P*. *penetrans*, 12,678 (53.4%) and 11,568 (48.7%) transcripts retrieved a significant blast hit (tBlastx with E-values < 1e^-5^ and bitscore above 50) against the transcriptomes of *P*. *coffeae* and *P*. *zeae*, respectively. A total of 9,935 transcripts were shared by these three species, while 9,404 (40%) being unique to *P*. *penetrans*. In the case of *P*. *thornei* and *P*. *vulnus* a tBlastx was performed against *P*. *penetrans*, as a lower number of sequences were available for these two species. About 41% (2,776 out of 6,667) and 30% (1,660 out of 5,630) sequences of *P*. *thornei* [11] and *P*. *vulnus*, respectively, showed a significant blast hit against *P*. *penetrans* transcriptome. In addition to an overall comparative analysis of the number of transcripts shared between *P*. *penetrans* and other *Pratylenchus* species, the most abundant transcripts and putative parasitism genes common to *Pratylenchus* are discussed in the following sections. We also used the previous EST data set generated for *P*. *penetrans* (n = 1,917), and tested against the full set of transcripts obtained in this study. A positive Blast hit was obtained to 81% (n = 1,552) of the ESTs against our *P*. *penetrans* transcriptome, with 92% of the transcripts showing a level of similarity above 90%. Taken together, these analyses suggest that the *P*. *penetrans* transcriptome produced here provides good representation of previous *P*. *penetrans* transcript data, yet has drastically expanded upon it to result in a large pool of novel transcripts not represented in the most closely related species (40%).

Gene Ontology (GO) was also performed to explore the hierarchical associations of *P*. *penetrans* transcripts by assigning a GO term and enzyme ontology (EC) (S1 Fig). From the total of 12,361 transcripts showing a significant BLAST hit to the NR database, 84% (n = 10,346) mapped to GO and/or EC terms, and could be annotated by biological process, molecular function, and/or cellular component. Additionally 360 transcripts were annotated with GO term through an InterProscan search, out of transcripts without a match to the NR or Swissprot protein databases. The majority of the transcripts represented in the biological process category are involved in embryonic development and in juvenile development, while in the molecular function category transcripts belonging to protein binding were the highest represented. At the cellular function the most prevalent categories were integral components of the membrane and nucleus.

### 3. An overview of the most abundant transcripts

To have a general overview of the most highly represented transcripts within the dataset, the RPKM values for the two nematode mRNA-Seq samples was used to identify the top 100 most abundant transcripts; 47 of which displayed no known annotation; roughly commensurate with the rest of the transcriptome. The remaining 53 annotated transcripts were distributed among different molecular functions, with most of them encoding housekeeping proteins, such as ribosomal proteins, heat shock proteins, or structural and regulatory components of the cuticle, and products which may bind cytoskeletal proteins such as collagen and actin. The top 20 most abundant transcripts of *P*. *penetrans* with annotation to the NR database are provided in Table 2, with the majority of them showing a positive match to sequences of the other four *Pratylenchus* species mentioned above. Like all nematode species, the life cycle of *P*. *penetrans* is punctuated by a molting process during the development of juveniles to adults. This process involves cell signaling within the hypodermis to prompt secretion of the new collagenous cuticle while shedding off the old cuticle. Collagens constitute a multigene family in nematodes [42], and the occurrence of these transcripts correlates well with the different development stages present in the pool of nematodes used for RNA extraction. Vitellogenin was also found among the highest expressed transcripts. This is a major yolk protein in many organisms, including nematodes [43], reflecting the presence of developing oocytes in females.

**Table 2:** List of the top 20 most abundant *Pratylenchus penetrans* transcripts with annotation to the NR protein database.

		*Pratylenchus* species								
TOP annotated transcript	FPKM	Pc	Pt	Pv	Pz	ANNOTATION—NR database	Top-Hit Species	E-value	Bit Score	Accession
1	11411.7	+	+	+	+	Actin	*Ditylenchus destructor*	0	788.10	ACT78498
2	4608.6	+	+	+	+	Protein bm210	*Brugia malayi*	2.63E-25	81.26	CDP91803
3	4037.0	+	+	+	+	Tropomyosin	*Heterodera glycines*	1.13E-128	384.40	AAQ12016
4	3959.7	+	+	+	+	Fatty acid and retinol binding partial	*Radopholus similis*	5.19E-93	285.03	AFI80890
5	3008.5	+	+	+	+	Vitellogenin—partial	*Ascaris suum*	0	634.02	ERG79133
6	2694.2	+	+	+	+	Polyadenylate-binding protein 1	*Loa loa*	0	702.98	XP_003136646
7	2621.6	+	+	+	+	Protein cpn-3	*Haemonchus contortus*	3.55E-67	217.62	CDJ87225
8	2319.4	+	+	+	+	Cre-pat-10 protein	*Meloidogyne incognita*	1.48E-106	316.24	AAW56830
9	2218.3	+	+	+	+	Protein tnt-2 isoform b	*Meloidogyne incognita*	3.34E-101	320.86	AFG25455
10	2181.7	+		+	+	Actin	*Caenorhabditis elegans*	1.21E-12	60.10	CAA34718
11	2114.3	+	+	+	+	Protein unc-87	*Heterodera glycines*	0	711.06	AAT70232
12	2056.7	+	+	+	+	Fatty acid desaturase domain containing protein	*Ditylenchus destructor*	4.55E-170	493.04	AGT39213
13	1896.2	+	+	+	+	Major sperm protein	*Meloidogyne hapla*	1.67E-82	256.53	CBA18117
14	1510.4	+	+		+	Heat shock protein 70	*Meloidogyne enterolobii*	0	175.64	AHG55039
15	1484.2	+	+		+	High mobility group protein	*Ascaris suum*	1.87E-24	103.22	ERG84055
16	1451.8	+	+	+	+	Ubiquitin extension protein	*Heterodera glycines*	1.82E-43	153.30	AAN32889
17	1359.4	+	+	+	+	Cre-mlc-3 protein	*Ancylostoma ceylanicum*	2.65E-83	259.61	EYB88032
18	1347.9	+	+	+	+	Translationally controlled tumor protein	*Ostertagia ostertagi*	7.56E-91	277.72	CAT00091
19	1317.0	+			+	Lea5 protein	*Steinernema carpocapsae*	4.27E-07	55.07	ABQ23240
20	1299.3	+			+	Cuticle collagen	*Ascaris suum*	1.59E-62	216.47	KHN84395

Pc: *Pratylenchus coffeae*; Pt: *Pratylenchus thornei*; Pv: *Pratylenchus vulnus*; Pz: *Pratylenchus zeae*.

Other interesting transcripts similar to neuromuscular function related genes, such as the FMRFamide-like peptides (*flp-1* and *flp-3*) and neuropeptide-like protein (*nlp-10*), were also prominently represented in this set. Using homology-based Blast searches we detected transcripts encoding up to 12, 9 and 3 members of the FMRFamide-like, NLP-like and insulin-like peptides families in the full dataset, respectively (S2 Table). Due to their broad functional scope, such as neuroendocrine and neuro-modulatory effects on locomotion, reproduction, feeding and behavior, there has been a long-standing interest in using the neuropeptidergic system and FLP-signaling pathways as alternative target for the development of novel methods to control animal- and plant-parasitic nematodes [44].

Transcripts with sequence identity to genes required for body wall muscle architecture and those related to nematode muscle contraction/locomotion (e.g. *unc-87* and *pat-10*) were among the top transcripts represented in this list. In *C*. *elegans* the *pat-10* gene encodes a body wall muscle troponin, which seems to be essential for muscle contraction and completion of embryonic morphogenesis and elongation, while *unc-87* gene is required for coordinated motility and normal muscle morphology [45]. Due to their significant involvement on nematode locomotion, locomotion-related genes have been considered for control measures against plant-parasitic nematodes, such as the use of RNAi-mediated gene silencing [37,38], and are of particular relevance to migratory parasites.

Interestingly, a transcript similar to a fatty acid- and retinoid-binding protein (homologue to other plant-parasitic nematodes FAR-1) was one of the most abundant transcripts in the dataset. FAR-1 is considered to encode a nematode secreted protein, which seems to be involved in host-nematode interaction of both animal- and plant-parasitic nematodes [46,47,48]. In animal-parasitic nematodes FAR proteins have been projected to facilitate nematode infection by scavenging and transporting fatty acids, required for developmental processes and cellular differentiation of the nematode, or playing a role in interfering with intracellular and intercellular lipid signaling related to host defenses [46,48]. In plant-parasitic nematodes they have been pointed out as a mediator of a complex manipulation of plant defenses responses against the invasion process of cyst and root-knot nematode species [47,49]. It will be interesting to validate whether FAR-1 will have similar functions on root lesion nematode species.

### 4. Silencing of *P*. *penetrans* genes through *in planta* expression of nematode dsRNA

RNAi interference represents a powerful technique for the analysis of gene function, and has shown promising results in the control of plant pathogens, including plant-parasitic nematodes [50]. We were able to identify for *P*. *penetrans* a meaningful number of transcripts encoding genes (33 out of 77) known to be involved in the RNAi pathway [36] of *C*. *elegans*, with the majority of them present as well in *P*. *coffeae* genome [13] (S3 Table). The RNAi-related transcripts distributed among four core functional groups, i.e. small RNA biosynthesis (n = 7), siRNA amplification proteins (n = 4), argonautes (n = 11) and RNA-induced silencing complex (n = 2), RNAi inhibitors (n = 2) and nuclear effectors (n = 7).

Induction of RNAi upon ingestion of double-stranded RNA (dsRNA) has proven to be effective in both *C*. *elegans* and in some plant-parasitic nematodes [51]. Because root lesion nematodes are migratory and dependent on their mobility, we conducted RNAi experiments against two genes related to locomotion and muscle architecture, i.e. *Pp-pat-10* (FPKM = 1,120 *in planta*) and *Pp-unc-87* (FPKM = 2,114 *in planta*), which were highly abundant among the nematode transcripts identified from infected roots at 3 and 7DAI. As RNAi-mediated nematode gene silencing that relied on soaking in double-stranded RNA solution has been established for these two genes in *P*. *coffeae* [37] and *P*. *thornei* [38], we evaluated the efficiency of silencing these nematode genes using the plant host for delivery of the nematode dsRNA fragments to generate transformed soybean hairy roots (S2A and S2B Fig).

**Fig 2:**
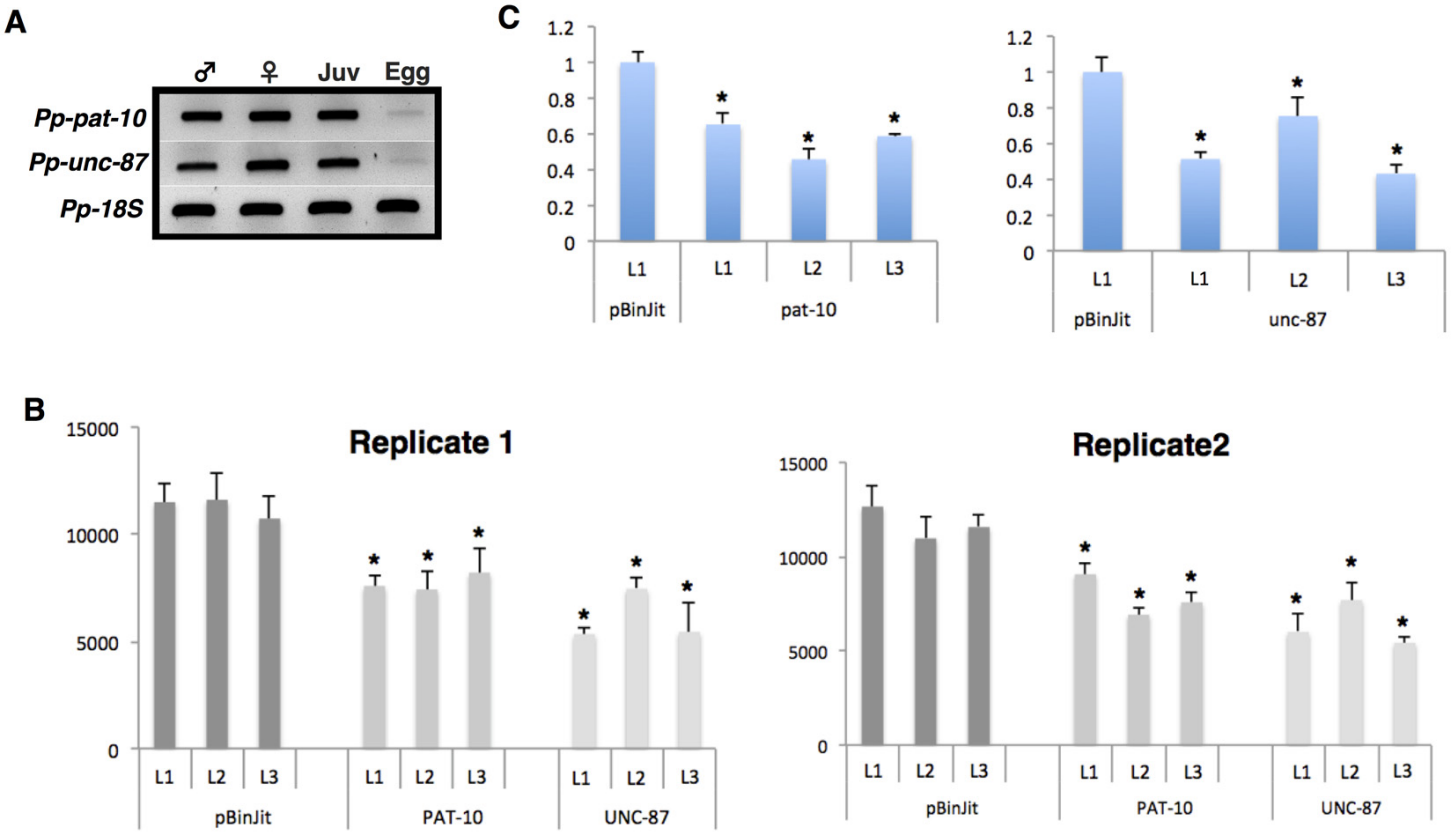
Effect of root RNAi-mediated silencing nematode genes on development and reproduction of *Pratylenchus penetrans*. (A) Semi-quantitative RT-PCR expression of *Pp-pat-10* and *Pp-unc-87* from mRNA of differential nematode stages (eggs, juveniles and adult stages). (B) Nematode challenge assays in transgenic soybean dsRNA hairy root lines 3 months after infection. Data shown represent the total mean number ± SEM of nematodes recovered from roots, using a pool of nine soybean hairy roots for each line. Replicate 1 and replicate 2 correspond to two independent biological assays, using the same hairy root lines. As control, hairy roots transformed with an empty vector (pBinJIT) were used. Asterisks denote statistically significant differences using Student’s t- test (P < 0.05). (C) Transcript levels of *P*. *penetrans* targeting genes that develop in the dsRNA expressing transgenic soybean hairy root lines. The expression levels were quantified as fold change values calculated by the 2^-ΔΔCt^ method, using *18S* rRNA gene as reference. Each bar represents the mean with standard error of two independent biological replicates, using three technical repetitions. Asterisks denote statistically significant differences using Student’s *t-*test (P < 0.05).

The amount of transcripts for the differential nematode developmental stages [eggs, juveniles (J2-J4), males and females] were initially evaluated using semi-quantitative RT-PCR. In general, a high transcript accumulation for both *Pp-pat-10* and *Pp-unc-87* was detected in all nematode stages (Fig 2A), except for eggs that showed a lower expression in comparison to the motile states. Three transgenic lines were then selected for each candidate based on the levels of expression of the pRAP17 vector intron (S2D and S2E Fig) and challenged with *P*. *penetrans*. Lines transformed with an empty vector (pBinJit) were used as control (S2C Fig). In comparison to control lines no apparent phenotypic variation could be observed between any of the dsRNA generated lines (data not shown).

To assess whether the *in planta* expression of dsRNA had any effect on nematode reproduction, the total number of nematodes supported by each line was evaluated three months after nematode inoculation. Two independent nematode challenge assays were performed and data are presented as the total average number of nematodes recovered from each line, in comparison to the nematode numbers obtained for three control lines (Fig 2B and 2C). In general we could observe a significant reduction (*P* < 0.05) of the total number of nematodes developing in hairy roots expressing dsRNA for each individually gene. In the case of *Pp-unc-87* lines a decrease up to 50% of the total number of nematodes could be observed, while for *Pp-pat-10* the best reduction percentage was about 40% fewer nematodes (*P* < 0.05) then the respective control lines (Fig 2B).

To assay the effect of each RNAi line on target gene abundance, total RNA was extracted from a pool of nematodes developing on these dsRNA hairy roots or control lines. Our results showed that the transcript abundance for each gene was significantly (*P* < 0.05) reduced on the nematodes collected, although showing different ranges of reduction depending on the different RNAi lines tested (Fig 2C). Collectively, our data demonstrate that plant-mediated silencing of selected nematode genes can confer some resistance to *P*. *penetrans* by reducing the total number of nematodes associated with such roots. However, *in planta* delivery, such as that carried out here, is less effective than targeting the same genes by dsRNA soaking [37,38] and perhaps provides a more pragmatic assessment of this technology for control. The observed differences are likely a consequence of silencing efficiency using dsRNA delivered by the plant *versus* soaking nematode experiments [37,38], as other studies have indicated different levels of variability in silencing efficacy among transgenic events, expression levels of dsRNA, and lines bearing the same type of construct [52]. Furthermore, as root lesion nematodes are migratory nematodes, able to move in and out of the roots, the continuous exposure of nematodes to dsRNA delivered by the plant can be arrested or reduced for the periods of time that the nematodes will exit the roots, as a consequential effect of the dsRNA exposure, or ordinary movements of the nematodes, may allow for some degree of recovery.

### 5. Screening for nematode proteins putatively involved in parasitism

To identify known nematode proteins with a role in parasitism, several comparisons were performed. First, the output results of the previous Blast strategies were searched for the presence of transcripts showing significant Blast hit to known parasitism-related proteins of other plant-parasitic nematodes, and secondly, Blast searches were performed using individual nematode sequences of particular interest, not necessarily present in the above databases, against the *P*. *penetrans* transcriptome.

To increase the likelihood of identifying further transcripts relevant for parasitism, an automatic ORF prediction was performed [31], revealing an ORF for 23,683 (out of 23,715) sequences. Predicted proteins were then analyzed for the presence of a predicted signal peptide, and the absence of a transmembrane domain (classical requirements for secretory proteins). Using only protein sequences starting with methionine, and a minimum length of 50 amino acids, 2,306 sequences were predicted to contain a signal peptide using SignalP4 [32]. Next, these predicted proteins were analyzed for transmembrane domains in order to remove sequences that were expected to be incorporated into the membrane of the nematode. 646 were predicted to contain one or more transmembrane domains, resulting in a final list of 1,660 transcripts encoding putatively secreted proteins, of which, 40% showed no similarity to known sequences deposited in either database used in this study. Given that most nematode parasitism-related genes described for other species are often associated with a non-annotated function or are specific to the Phylum Nematoda [30], some of these transcripts could be relevant to the host-nematode interaction. Given that not all secreted proteins are directly involved in parasitism, further analysis of these transcripts will therefore be required.

#### 5.a. Cell wall-modifying enzymes (CWME) and other CAZys

The plant cell wall constitutes the primary barrier faced by most plant-parasitic nematodes and the production of enzymes capable of breaking down the cell wall is a critical step for successful parasitism. Plant-parasitic nematodes often secrete a different range of CWMEs, mostly acquired by horizontal gene transfer from bacteria [53]. The expression of such enzymes appears to be closely associated with disassembly and modification of the host cell wall, involved during host invasion, and the migratory process of the motile nematode stages [53,54]. They may also be involved in softening the host cell walls to allow the nematode stylet to feed from the punctured cells [4].

*Pratylenchus penetrans* transcripts showing high similarity to putatively secreted CWME distributed among different families (Table 3) include endo-1,4-β-glucanases (GH5), pectate lyases (PL3), xylanases (GH30), polygalacturonases (GH28) and arabinogalactan endo-1,4-β-galactosidase (GH53). As previously shown for other *Pratylenchus* species (*P*. *coffeae*, *P*. *thornei* and *P*. *zeae*), one of the most represented nematode CWMEs identified belong to the GH5 family (endo-1,4-β-glucanases), with some of the transcripts harbouring a predicted signal peptide and a carbohydrate-binding module (CBM2). Some of them displayed great similarity to previously described *P*. *penetrans* endo-1,4-β-glucanases (e.g. *Pp-ENG-1* and *Pp-ENG-2*), while others showed high similarity to other nematode species, including both sedentary (e.g. *Heterodera* and *Globodera* spp.) and migratory plant-parasitic nematode species (e.g. *Radopholus similis*), supporting the previous idea that *P*. *penetrans* harbours different gene members of this family [55]. We identified five transcripts of the PL3 family (pectate lyases) showing highest similarity to cyst or root-knot nematodes (Table 3). In both cases, some transcripts displayed the presence of a signal peptide and absence of a transmembrane domain. Three transcripts with similarity to a xylanase protein identified from another migratory plant-parasitic nematode, of the same family as *Pratylenchus*, *Radopholus similis* [56], also contained a putative signal peptide for secretion and no transmembrane domain. This type of protein has been shown to play a significant role in the virulence of a fungus (*Magnaporthe oryzae* Cavara) affecting both penetration and expansion of this species in infected plants [57]. Four polygalacturonase transcripts were identified from the family GH28, with the most similar sequence identified from *Meloidogyne incognita*, although no signal peptide could be detected among these different transcripts. A single transcript encoding for an arabinogalactan endo-1,4-β-galactosidase (GH53 family), thought to be specific to cyst nematodes [53], has been reported for *P*. *coffeae* [10] and also found in the transcriptome of *P*. *penetrans*. Crucially, none of the cell wall degrading enzyme here have higher similarity to bacterial or fungal sequences than to other nematodes species, suggesting they do not arise from contamination.

**Table 3:** Overview of putative cell wall modifying enzymes identified within the *Pratylenchus penetrans* transcriptome.

Enzyme	Putative function	*P*. *penetrans*			
		Number of transcripts	Best min. E-value	Best species hit	Sequence ID
**Endo-1,4-β-glucanase (GH5)**	Plant cell wall degradation	21 (SP)	1.74E-90	*Pratylenchus penetrans*	BAB68523
**Expansin-like protein**	Plant cell wall extension	7 (SP)	4.04E-42	*Heterodera glycines*	ADL29728
**Pectate lyase (PL3)**	Plant cell wall degradation	5 (SP)	9.28E-84	*Heterodera glycines*	ADW77534
**Polygalacturonase (GH28)**	Plant cell wall degradation	4	1.06E-58	*Meloidogyne incognita*	AAM28240
**Xylanase (GH30)**	Plant cell wall degradation	3 (SP)	0	*Radopholus similis*	ABZ78968
**Arabinogalactan endo-1,4-beta-galactosidase (GH53)**	Plant cell wall degradation	1 (SP)	7.00E-120	*Heterodera glycines*	ACY02856

SP: Indicates that at least one sequence of each respective family of enzymes was predicted to have a putative signal peptide.

In addition, other *P*. *penetrans* transcripts were found with high similarity to other carbohydrate active enzymes (CAZy) when searched using the CAZymes Analysis toolkit [33]. S4 Table lists other predicted CAZy proteins in the *P*. *penetrans* transcriptome, with 17 identified as glycoside hydrolases (GH), 2 encoding carbohydrate esterases (CE), and 30 glycosyl transferase (GT) proteins. Previously sequences with similarity to bacteria GH16 (beta-1,3-endoglucanase) proteins, which occur mainly on the fungal feeder *B*. *xylophilus*, were also reported for *P*. *coffeae* [10], however, within the generated transcriptome no transcripts of this family were identified.

Transcripts encoding expansin-like proteins with greatest similarity to *Heterodera avenae* Wollenweber, 1924 and *H*. *glycines* were also detected among the *P*. *penetrans* transcriptome (Table 3). These types of proteins play an important role in physiological processes requiring cell wall modifications and can loosen and disrupt the hydrogen-bonding networks of cell wall polysaccharides without hydrolyzing them [58].

#### 5.b. Detection of other known nematode proteins putatively involved in parasitism

Transcripts showing similarity to nematode proteins with various putative roles in parasitism are presented in Table 4. The most highly represented group of transcripts encoded for transthyretin-like proteins, with great similarity to both plant- and animal-parasitic nematodes. The majority of these sequences are predicted to encode proteins with a signal peptide, suggesting a potential secretion of these proteins. They constitute a highly specific and conserved family within the Phylum Nematoda, and are characterized by the presence of a transthyretin-like domain (PF01060; IPR001534; DUF290), which is one of the most abundant nematode-specific domains [59]. Although the molecular function/s of this family of genes is/are still not clear, they have been reported for both sedentary and migratory species with different members of this family showing a distinct spatial distribution, e.g. pharyngeal gland cell in the case of *Xiphinema index* Thorne and Allen, 1950 [60], or in the ventral cord or tissues surrounding the vulva in the case of *Radopholus similis* [61]. In animal-parasitic species they have been detected in the excretory-secretory (ES) products of several species [62,63].

**Table 4:** Output results of Blast searches using known nematode proteins with a putative function in parasitism reported for other plant-parasitic nematode species, and found in *Pratylenchus penetrans* transcriptome.

Descriptions / Gene product	*P*. *penetrans*					Reference
	Transcripts	Best e-Value	Best % Identity	Species	Accession number	
**14-3-3 b**	2	2.12E-166	98.28	*Meloidogyne incognita*	AAR85527	[76]
**Acid phosphatase**	3 (SP)	4.76E-173	56.97	*Meloidogyne incognita*	AAN08587	[77]
**Annexin**	1	2.43E-160	67.56	*Heterodera schachtii*	AAN32888	[78]
**Calreticulin**	1 (SP)	6.69E-170	81.66	*Meloidogyne incognita*	AAL40720	[79]
**FAR-1**	2 (SP)	2.97E-85	83.15	*Globodera pallida*	CAA70477	[47]
**Galectin**	2	3.06E-68	63.38	*Globodera rostochiensis*	AAB61596	[80]
**Glutathione peroxidase**	3 (SP)	1.29E-138	83.90	*Globodera rostochiensis*	CAD38523	[81]
**Glutathione-S-transferase**	10 (SP)	1.03E-70	55.94	*Meloidogyne incognita*	ABN64198	[80]
**Peroxiredoxin**	4	4.07E-118	90.31	*Globodera rostochiensis*	CAB48391	[82]
**SXP/RAL-2**	1 (SP)	2.60E-37	57.69	*Meloidogyne incognita*	AAR35032	[83]
**Transthyretin-like proteins**	33 (SP)	2.41E-62	86.15	*Radopholus similis*	CAM84510	[61]
**VAP-1 (Venom allergen-like protein)**	3 (SP)	4.82E-84	67.58	*Globodera rostochiensis*	AEL16453	[70]

SP: Indicates that at least one sequence of each respective family of enzymes was predicted to have a putative signal peptide.

Other nematode proteins of interest occurring in both migratory or sedentary nematode species were found in *P*. *penetrans*, such as 14-3-3 proteins, acid phosphatase, annexin-like, calreticulin, FAR-1, galectin, SXP-RAL-2, and venom allergen-like proteins (VAP-1). While transcripts encoding SPRY and Ubiquitin domains were also detected, these did not encode SPRYSECs or Ubiquitin extension proteins. It is important to note that SPRYs and Ubiquitins are extremely common in the animal kingdom, and only a subset of which are deployed as parasitism genes and are easily identified by unique characteristics [64]. Noteworthy to mention is that several transcripts showed similarity to putative secreted proteins isolated from the secretory glands of both *Heterodera glycines* and *Meloidogyne incognita* deposited in both EST nematode databases (S5 Table). We detected a partial putative chorismate mutase, which has also been reported in other Pratylenchidae species, such as *P*. *coffeae* [10], *P*. *thornei* [11], and *Hirschmanniella oryzae* [65], but absent in *P*. *zeae* transcriptome [13]. An InterPro scan search for conserved domains suggested the presence of a chorismate mutase type 2 domain (IPR020822; Pfam01817). When querying *P*. *penetrans* predicted chorismate mutase type 2 domain against the NCBI non-redundant protein database (NR), the highest similarity sequences returned are from several cyst nematodes (*Globodera* spp.), followed by other plant-pathogenic bacteria, such as *Ralstonia solanacearum* (S6 Table).

There is increased evidence that plant-parasitic nematodes harbor a significant number of genes involved in protection from the host defenses, such as reactive oxygen species (ROS) and xenobiotic compounds [24,66]. Consistent with this, several transcripts encoding catalase, thioredoxin, peroxiredoxin, superoxide dismutase, glutathione synthetase, glutathione peroxidase and glutathione-S-transferase were identified, with some of them showing significant similarity to proteins with a parasitism role in other plant-parasitic species (Table 3). Plants respond to nematode infection by the production of reactive oxygen species (ROS), which are toxic to the nematode, and contribute to strengthening the cell wall, as well as prompting the activation of other defense responses from the host [67]. On the other side, nematodes possess a repertoire of enzymes for scavenging ROS, including catalase, peroxidase and superoxide dismutase, that are assumed to protect the nematode from the damaging effects of ROS, or alternatively to have a direct effect on the regulation of the host defenses and to modulate ROS-mediated defense signaling [67].

A significant number of transcripts belonging to diverse classes of putatively secreted proteases by *P*. *penetrans* (S7 Table) were also represented (e.g. aspartyl protease, cathepsin proteases, metalloproteases and serine-peptidases). The most represented families were C01A (15 transcripts), S01A (44 transcripts), M12A (6 transcripts) and S33 (5 transcripts). Proteases are ubiquitous proteolytic enzymes that cleave internal peptide bonds of proteins and peptides. This group of proteins is distributed in a diverse range of organisms including bacteria, plants, vertebrates, and abundantly present as well in invertebrates, such as nematode species. In the case of animal-parasitic nematodes, there are many examples of secreted peptidases that are involved in host-parasite interactions, for invasion and migration of host tissues, nutrition, or blocking immune responses of the host [68]. In plant-parasitic nematodes some of these secreted proteases have been localized in the nematodes’ sub-ventral glands and secreted into the plant apoplasm by *M*. *incognita* [69], suggesting their participation in parasitism by softening or destroying plant cell walls during migration, pre-digesting nutrients in sedentary stages, or inactivating plant defense proteins.

### 6. Validation and expression of a selected set of nematode transcripts

To validate our *de novo* assembly prediction of transcripts encoding particular nematode genes, semi-quantitative RT-PCR analyses were conducted for 11 gene targets that spanned a range of differential expression, and included different nematode predicted functions. In this selection, we included transcript sequences similar to genes encoding cell wall-degrading enzymes (two endo-1,4-β-glucanases and one pectate lyase), nematode detoxification machinery (a glutathione peroxidase, a glutathione-S-transferase and a catalase), predicted transcripts encoding a FAR-1 and a venom allergen-like protein (VAP-1), which have been shown to be implicated in the host defense suppression of root-knot and cyst nematodes respectively [49,70], as well as transcripts encoding a peptidase C1A and two pioneer candidate genes.

**Fig 3:**
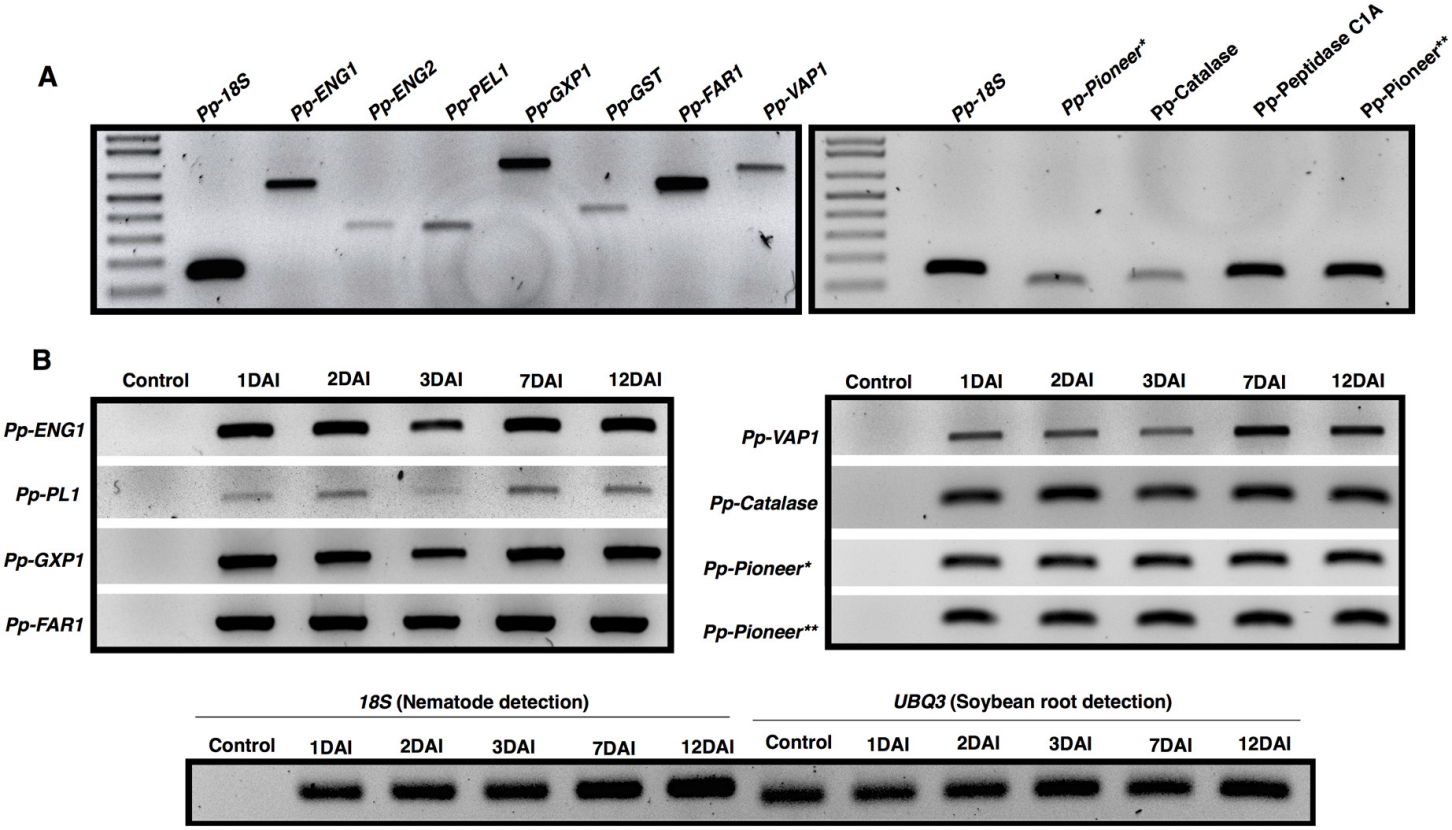
Semi-quantitative RT-PCR showing the transcript levels for a subset of predicted *Pratylenchus penetrans* transcripts. (A) RT-PCR amplification was performed using the generated cDNA from total RNA collected from a pool of mixed stages (eggs, juveniles and adult stages) of *P*. *penetrans*. (B) Upper panel: detection of a subset of *P*. *penetrans* genes putatively involved in parasitism using total RNA extracted from nematode infected soybean roots, at different days after nematode infection (1, 2, 3, 7 and 12 days after infection). Lower panel: the nematode *18S* rDNA gene was used as control to validate the presence of *P*. *penetrans* in the infected roots, while *UBQ3* gene was used to validate plant gene detection. Control corresponds to non-infected soybean roots.

The transcript level of each selected candidate was first evaluated using cDNA obtained from total RNA extracted from a pool of nematodes containing mixed stages (eggs, juveniles and adults) (Fig 3A). All predicted genes could be amplified, many with varying abundance. Transcripts similar to genes encoding for *FAR-1*, *ENG1*, *GXP1*, *peptidase C1A* and a pioneer gene were amongst the most highly expressed within this subset. To confirm that putative parasitism genes in the mixed stage transcriptome are indeed expressed during plant-nematode interaction, semi-quantitative RT-PCR was carried out for a subset of genes at different time points after nematode infection. Using total RNA extracted from nematode-infected soybean roots 1, 2, 3, 7 and 12 DAI, evidence of expression is found for all tested putative parasitism genes, consistent with their hypothesized role (Fig 3B). In general, an accumulation of transcripts for each gene could be noticed 1 to 12 days after nematode infection, which appears to correlate with a high number of nematodes established within the root tissues, as indicated by an increase in control gene transcript abundance.

### 7. Identification of *P*. *penetrans* transcripts upregulated while *in planta*

In this study we also characterized *P*. *penetrans* transcripts obtained from nematode soybean infected roots generated in parallel to this work (Vieira *et al*. unpublished). While the proportion of nematode reads obtained from the infected root tissues increased from 3 to 7DAI, consistent with a higher number of nematodes infecting the roots, as expected the vast majority of reads were of plant origin. Of the reads from the four libraries of *in planta* mRNA-seq, which did not map to soybean genome, between 280,312 and 1,128,345 reads mapped to *P*. *penetrans* transcriptome. The unmapped reads that mapped back to the *P*. *penetrans* assembly were deposited under BioProject ID PRJNA304159, and served two-fold: firstly, to provide additional evidence that those transcripts identified as putative parasitism genes (section 5 above) are indeed expressed when the nematode is in the plant; secondly, to identify genes specifically upregulated while *in planta*. In general, no difference was detected between 3, and 7 DAI and so these are considered together as *in planta*.

**Fig 4:**
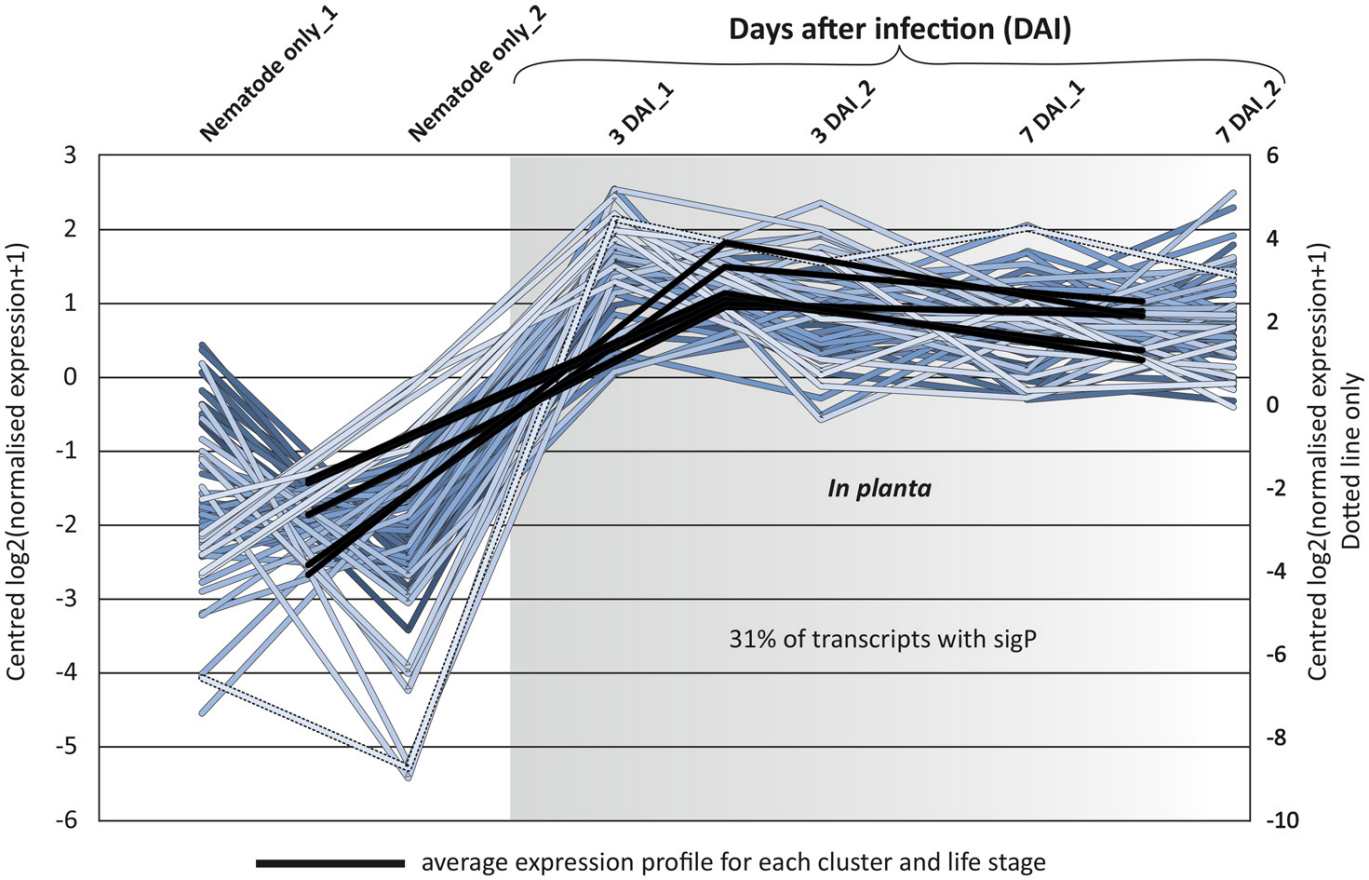
Identification of *Pratylenchus penetrans* transcripts significantly upregulated *in planta*. Differential expression analysis was carried out on all transcripts in the *P*. *penetrans* transcriptome. Those differentially expressed at a significant level (FDR *p* < 0.001, min fold change 2) were grouped into various expression clusters. Here, 5 clusters, each with variations in magnitude of the same biologically relevant expression profile contains a total of 58 trinity components. The average for each cluster and each life stage is indicated by black bars. Of these transcripts upregulated, 31% encode proteins with putative signal peptides for secretion; a 3.5 fold enrichment over the rest of the transcriptome.

Using the total FPKM average of the soybean-infected roots, the most highly represented nematode transcripts *in planta* have similarity to metabolic or nematode developmental genes, including vitellogenin (FPKM = 10,915), actin (FPKM = 8,952) and cuticle collagens (FPKM = 1,899), with a hypothetical protein Bm1 of *Brugia malayi* on the top of these transcripts (FPKM = 26,713) (Table 5). Although, most of these transcripts are present in other root lesion species, others seemed to be specific to *P*. *penetrans*, as no significant similarity was found within sequences of other *Pratylenchus* species herein compared. Importantly, most of the known nematode secreted proteins identified above, and putatively involved in parasitism, (e.g. calreticulin, cell wall degrading enzymes, FAR-1, VAP-1), show evidence of expression *in planta*, with some of them placed within the top 20 most abundant transcripts, such as transcripts with similarity to FAR-1, calreticulin and various proteases (Table 5), supporting their identification and independent validation by the semi-quantitative RT-PCR (Fig 4).

**Table 5:** List of the top 20 most abundant *Pratylenchus penetrans* transcripts identified in nematode infected soybean roots (top), the top 20 most abundant *P*. *penetrans* transcripts identified in nematode infected soybean roots which encode a putative signal peptide (bottom).

		*Pratylenchus* species								
Transcript	FPKM Planta	Pc	Pt	Pv	Pz	ANNOTATION—NR Database	Top-Hit Species	Best e-value	Bit Score	Accession
Top 20 of all transcripts found in *planta*										
1	26,730.55	+	+	+	+	Hypotethical protein Bm1	*Brugia malayi*	3.04E-21	89.35	XP_001895031
2	10,915.12	+	+	+	+	Vitellogenin—partial	*Ascaris suum*	0	634.02	ERG79133
3	8,952.19	+	+	+	+	Actin	*Ditylenchus destructor*	0	788.10	ACT78498
4	3,525.76					No match				
5	3,501.78	+	+	+	+	Fatty acid and retinol binding partial	*Radopholus similis*	5.19E-93	285.03	AFI80890
6	3,370.08	+	+	+	+	Fatty acid desaturase domain containing protein	*Ditylenchus destructor*	4.55E-170	493.04	AGT39213
7	3,227.35					No match				
8	2,471.04					No match				
9	2,445.49	+		+	+	Actin	*Caenorhabditis elegans*	1.21E-12	60.10	CAA34718
10	2,267.89					No match				
11	2,030.17	+	+	+	+	Tropomyosin	*Heterodera glycines*	1.13E-128	384.80	AAQ12016
12	1,899.02				+	Cuticular collagen	*Meloidogyne incognita*	1.24E-31	132.11	AAB63467
13	1,723.74					No match				
14	1,626.76	+	+	+	+	Polyadenylate-binding protein 1	*Loa loa*	0	702.98	XP_003136646
15	1,479.70				+	No match				
16	1,374.32				+	No match				
17	1,344.32	+			+	Cuticle collagen 6	*Ascaris suum*	2.91E-19	98.21	ERG81835
18	1,308.45	+	+	+	+	Protein unc-87	*Heterodera glycines*	0	711.06	AAT70232
19	1,268.15					No match				
20	1,228.26	+	+	+	+	Fatty acid elongation protein 3	*Loa loa*	7.65E-102	313.54	XP_003148091
Top 20 transcripts with signal peptide *in planta*										
		***Pratylenchus* species**								
		**Pc**	**Pt**	**Pv**	**Pz**					
1	10,915.12	+	+	+	+	Vitellogenin—partial	*Ascaris suum*	0	634.02	ERG79133
2	3,501.78	+	+	+	+	Fatty acid and retinol binding partial	*Radopholus similis*	5.19E-93	285.03	AFI80890
3	1,899.02				+	Cuticular collagen	*Meloidogyne incognita*	1.24E-31	132.10	AAB63467
4	1,052.87	+	+	+		Hypothetical protein Y032_0510g2735	*Ancylostoma ceylanicum*	6.73E-19	88.96	EYC42944
5	836.70	+	+	+	+	Cathepsin l-like cysteine proteinase	*Ditylenchus destructor*	1.66E-155	459.14	ACT35690
6	834.28					No match				
7	772.08					No match				
8	735.92	+	+	+	+	Cathepsin l	*Ancylostoma ceylanicum*	1.68E-89	287.34	EYC42688
9	689.63	+			+	Catalase	*Ditylenchus destructor*	0	624.39	AFJ15102
10	686.05				+	Nematode cuticle collagen domain protein	*Ancylostoma ceylanicum*	2.29E-29	120.55	EYC31354
11	642.97	+	+	+	+	Protein disulfide isomerase	*Ancylostoma ceylanicum*	0	727.62	EYB93146
12	609.44	+	+	+	+	Peptidase c1a domain containing protein	*Onchocerca volvulus*	2.75E-143	423.70	AAC47348
13	607.27	+	+	+		Unnamed protein product	*Haemonchus contortus*	2.90E-20	93.58	CDJ86795
14	602.80	+			+	Protein isoform b	*Oesophagostomum dentatum*	3.32E-15	81.26	KHJ85643
15	599.83	+	+			No match				
16	588.96	+	+	+	+	Protein BM-CNB-1	*Brugia malayi*	7.12E-06	51.62	CDP98667
17	581.96	+	+	+	+	Calreticulin	*Pratylenchus goodeyi*	0	528.86	AIW66697
18	573.84					No match				
19	569.85	+	+			Neuroendocrine convertase 2	*Heterodera glycines*	0	1177.54	AAK66762
20	554.77					No match				

Pc: *Pratylenchus coffeae*; Pt: *Pratylenchus thornei*; Pv: *Pratylenchus vulnus*; Pz: *Pratylenchus zeae*.

Secondly, in an attempt to identify transcripts integral to *P*. *penetrans* infection without *a priori* knowledge of sequence, those transcripts specifically up-regulated *in planta* (FDR p < 0.001, min fold change 2) were identified and grouped into expression clusters (Fig 4). This has identified 58 trinity components (66 transcripts), as specifically upregulated *in planta*. Within this expression cluster, known effectors were re-identified. Further, 31% of the transcripts up-regulated *in planta* encoded proteins with a predicted signal peptide for secretion–representing an over 3.5 fold enrichment compared to the rest of the transcriptome. Given that all proteins directly involved in the plant-nematode interaction must be secreted, together the enrichment for signal peptides and the re-identification of known effectors gives confidence in the approach to identify genes of importance during parasitism. Of the annotated proteins, peptidases (mainly cathepsin) and peptidase inhibitors were strongly represented; supporting a possible role in developmental processes (e.g. nutrition) or specific interaction with the host [71]. In animal parasitic nematodes proteases and protease inhibitors are often upregulated during early infection [68,72]. Secreted proteases have been suggested as associated with digestion of host proteins, but also in the inactivation of proteins of the host’s immune system [73], while secreted protease inhibitors could be related to suppression of the host immunity [74]. Likewise, a cysteine proteinase C1A as been found expressed in the gland cells of the migratory pinewood nematode (*B*. *xylophilus*), suggestive of putative function in digestion of host tissues during migration, or targeting of host proteins involved in defenses responses [66].

Interestingly however, the majority of sequences present in the expression cluster (~65%) are pioneers, furthermore, most of these genes appear to be specific to RLN, as no homologues were found in other nematode species. Together, this highlights how little is still known about those genes utilized by the nematode during infection, and sheds light on potential novel genes involved in the establishment of the nematode within the roots. Given that the *in planta* samples typically contained >95% plant transcripts, the coverage for nematode transcripts was on average low. The result is that only those transcripts, and in particular those differentially expressed, of the highest magnitude and consistency will be detected. Nevertheless, by their very nature, these detected transcripts will be of high confidence, yet, probably only representing the upper most fraction of those integral to earlier infection time periods. Putatively secreted, upregulated *in planta*, pioneer genes will be high priority targets for future molecular study, as some of them seemed to be specific to *P*. *penetrans*, in particular as targets for host delivered RNAi, as described for *P*. *penetrans* here.

## Conclusions

The past years have witnessed an increase in the number of genomic and transcriptome studies of animal- and plant-parasitic nematodes. Despite the economic importance caused by *P*. *penetrans*, up to date little was known about the molecular basis of this species. Herein we provide a characterization for *P*. *penetrans* transcriptome generated by Illumina sequencing, including the detection and evaluation of nematode transcripts detected at early time points after nematode infection in soybean roots. The generated data in this study represent a substantial expansion of the transcriptome resources available for this species, including a preview into the molecular actors ensuing such plant-nematode interactions.

In general, the transcriptome of *P*. *penetrans* seems to follow common features reported for other root lesion species (*P*. *coffeae*, *P*. *thornei* and *P*. *zeae*). As found for these species, *P*. *penetrans* displays a similar range of cell wall degrading enzymes, showing a much reduced number of such enzymes in comparison to other more specialized sedentary plant-parasitic species, such as cyst, root-knot and the false root-knot nematode species [24,54,75]. As in other plant parasitic species, our Blast analyses indicate that a significant number of candidate genes lack annotation and a predicted function. Focusing on predicted nematode secreted proteins found in this transcriptome, we observed specific members to be up-regulated at the early time points of infection. In the present study, we observed an enrichment of predicted secreted proteins along the early time points of parasitism by this species. The representative list of genes examined using RT-PCR confirms their expression during the host infection. The expression patterns of the different candidate genes raise the possibility that they might be involved in critical steps of *P*. *penetrans* parasitism. These results will ultimately help elucidate the mechanisms underlying the pathogenicity of *P*. *penetrans*, as well as providing the baseline for selection of future nematode gene candidates with a potential role on parasitism, ultimately to design effective control strategies against root lesion disease caused by this broad host range pathogen.

## Supporting Information

S1 Fig Distribution of *Pratylenchus penetrans* transcripts assigned to a Gene Ontology (GO) term predicted by Blast2Go software. (TIFF)

S2 Fig Gene cloning and transformation of soybean hairy roots for host-derived RNA-interference of *Pratylenchus penetrans* genes. (TIFF)

S1 Table List of primers. (PDF)

S2 Table Transcripts of *Pratylenchus penetrans* encoding predicted neuropeptides. (PDF)

S3 Table *Pratylenchus penetrans* transcripts encoding for genes involved in the RNAi pathway, based upon the genes identified for *Caenorhabditis elegans* [36]. (PDF)

S4 Table List of other carbohydrate active enzymes identified in *Pratylenchus penetrans* transcriptome. (PDF)

S5 Table Significant Blast search results against putative esophageal gland cell secretory proteins of *Meloidogyne incognita* (root-knot nematode) and *Heterodera glycines* (soybean cyst nematode). (PDF)

S6 Table The top best hits of the Blastp search against non-redundant protein database at the NCBI using *Pratylenchus penetrans* chorismate mutase type 2 domain sequence as query. (PDF)

S7 Table Summary of transcripts encoding proteases with a putative signal peptide and no TMHMM found in *Pratylenchus penetrans* transcriptome. (PDF)
