# Supplementary figures and images for: The Pratylenchus penetrans Transcriptome as a Source for the Development of Alternative Control Strategies: Mining for Putative Genes Involved in Parasitism and Evaluation of in planta RNAi
Source: PLoS One. 2015 Dec 14;10(12):e0144674. doi: 10.1371/journal.pone.0144674 (PMC4684371; doi:10.1371/journal.pone.0144674)

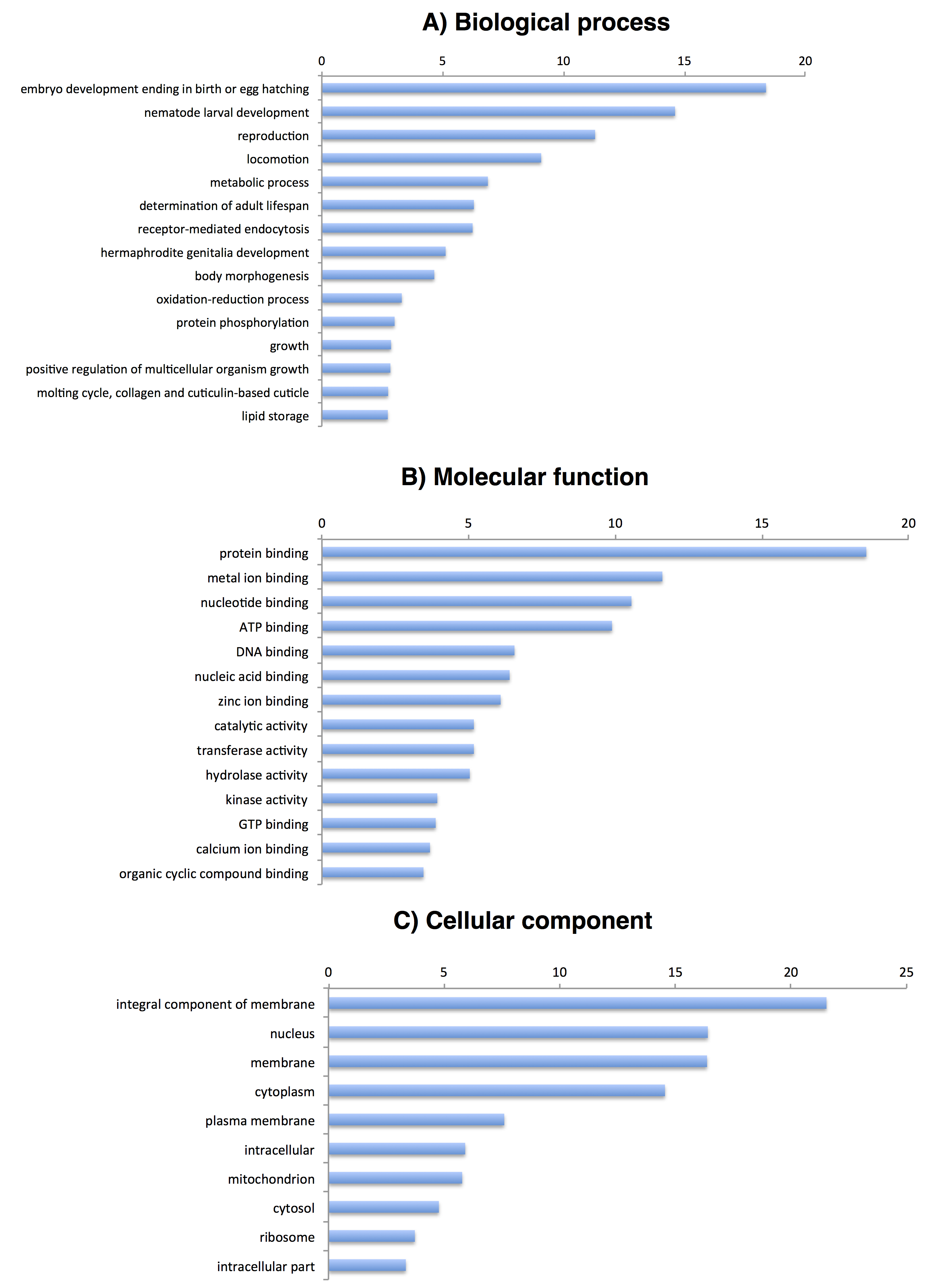

Supplement: S1 Fig — (TIFF) [file pone.0144674.s001.tiff]

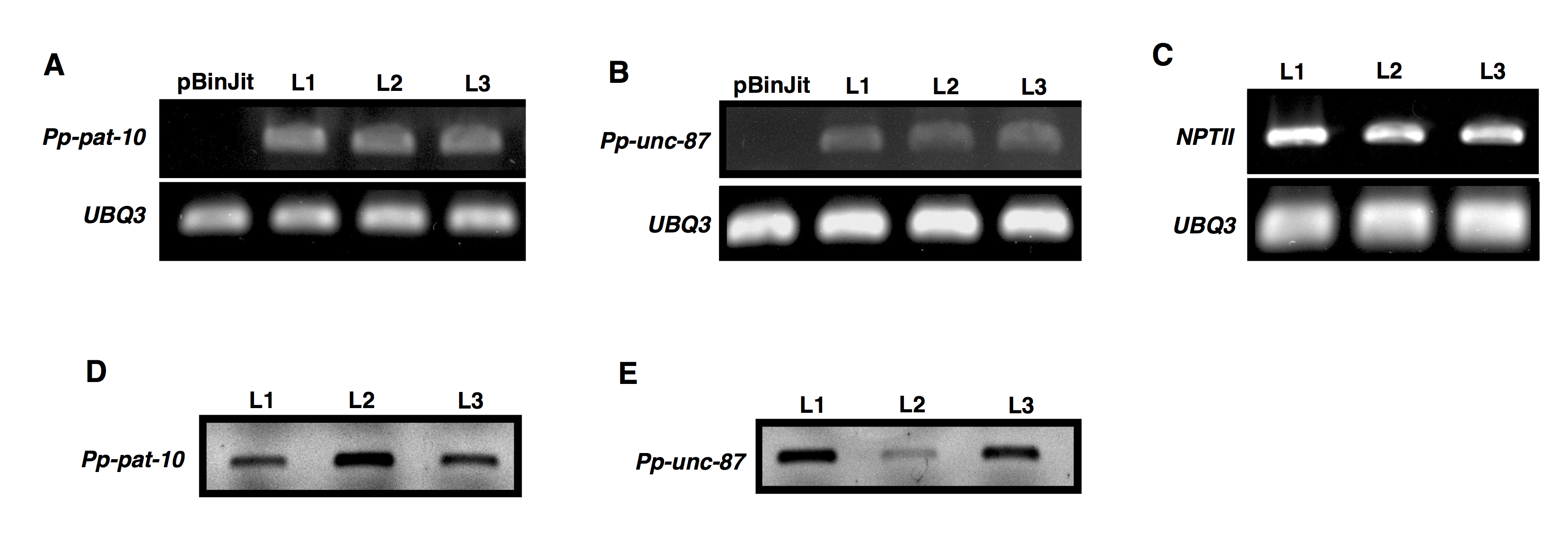

Supplement: S2 Fig — (TIFF) [file pone.0144674.s002.tiff]
