# Supplementary material for: The Pratylenchus penetrans Transcriptome as a Source for the Development of Alternative Control Strategies: Mining for Putative Genes Involved in Parasitism and Evaluation of in planta RNAi
Source: PLoS One. 2015 Dec 14;10(12):e0144674. doi: 10.1371/journal.pone.0144674 (PMC4684371; doi:10.1371/journal.pone.0144674)
Supplement: S1 Table — (PDF) [file pone.0144674.s003.pdf]

S1 Table. List of primers.

Primers used for the pRAP17 constructs	
**PCR amplification**	
Pp-pat-10 - F	CACCGTTTGACCGAGGCAAGAATG
Pp-pat-10 - R	GTCACCGGCCATCAGTT
Pp-unc-87 - F	CACCGTGACTCCCAGAAGTTGATGAC
Pp-unc-87 - R	TTGTTTGTACCCGACTGGAG
NPT-II - F	TCGGCTATGACTGGGCACAACAGA
NPT-II - R	AAGAAGGCGATAGAAGGCGATGCG
FMV - F	AAGAAGCCCTCCAGCTTCAAAG
Intron 2 - R	CCAGTTAACGTGTCTCATAT
eGFP - F	ATGGTGAGCAAGGGCGAGGAGC
eGFP - R	TCGTCCATGCCGAGAGTGATCCCG
Ri K599 - F	TCAGCCTCCCCGCCGGATG
Ri K599 - R	ATGCAAAAGACAGGATTGATCGCA
**RT-PCR reaction**	
Pp-pat-10 - F	GAATTCTGCGCACTGGTCTA
Pp-pat-10 - R	TGCCTCTTTCAGCTCCTTTC
Pp-unc-87 - F	CCCGTAACACAACAACAAAGG
Pp-unc-87 - R	TCGATTTGTACCGGACTGAAG
Intron 2 - F	CTGACCTGCAAACACGTTAAAT
Intron 2 - R	TTCATTGAATTTGATGGCCATAGG

Primers used for nematode candidate genes (RT-PCR)	
Pp-ENG1-F	GCAACAATCCGCACATCATC
Pp-ENG1-R	CTGGTGAACCGGAACAACT
Pp-ENG2-F	GGATTTGCGCAACAAAGCG
Pp-ENG2-R	ATCATCATCATCAGCCATCCAA
Pp-PEL1-F	GAGAAGTTCAGCCGAGTGTATC
Pp_PEL1-R	GACTTGATGCTAATTGCCGATTT
Pp-GXP1-F	GGTCCACCGAAACAGGAAA
Pp-GXP1-R	TGGTGCAGTGAGCCATTT
Pp-GST - F	CGGAAAGGACGAATGGGAAA
Pp-GST - R	CTTCCGGGCAGCCAAATA
Pp-FAR1-F	TGGCCTTTATAGCCATAGCAC
Pp-FAR1-R	GCCTTCGGGTTTCAGCA
Pp-VAP1-F	CGGGCCGAAACCTAATACAA
Pp-VAP1-R	GGCACAATGCAAAGCGAAT
Pp-Pioneer* - F	TTTCTTCCACTCTGCGATG
Pp-Pioneer* - R	CCCAAACATATTTATCCTTCTTCTC
Pp-Catalase - F	CCAACATGGTCTGGGACTTT
Pp-Catalase - R	GACCCGAATCCGTCCATTT
Pp-peptidase C1A - F	TGACCGGAACCAACACATTC
Pp-peptidase C1A - R	GACCTCCTGAACCGACAAATAG
Pp-Pioneer** - F	TACGGTTATAATCCGCCTCCT
Pp-Pioneer** - R	CCCATTCCACCACCAAATGA
Pp-18S-F	ATGAGAGGGCAAGTCTGGTG
Pp-18S-R	GAAAGCCGAAGACATCCAGT
Gmax-Ubi3-F	GTGTAATGTTGGATGTGTTCCC
Gmax-Ubi3-R	ACACAATTGAGTTCAACACAAACCG
