# Supplementary material for: The Pratylenchus penetrans Transcriptome as a Source for the Development of Alternative Control Strategies: Mining for Putative Genes Involved in Parasitism and Evaluation of in planta RNAi
Source: PLoS One. 2015 Dec 14;10(12):e0144674. doi: 10.1371/journal.pone.0144674 (PMC4684371; doi:10.1371/journal.pone.0144674)
Supplement: S2 Table — (PDF) [file pone.0144674.s004.pdf]

S2 Table. Transcripts of *Pratylenchus penetrans* encoding predicted neuropeptides. Blastx searches (E-value < 1e-5) were performed against the different neuropeptide homologues described for *Caenorhabditis elegans.*

Predicted transcripts encoding FMRFamide-like peptides*

		Pratylenchus penetrans		
**FLPs**	*C. elegans*	# of transcripts	E-value	High bitscore
flp-1	F23B2.5	1	6.47776E-11	60.08
flp-3	W07E11.2	1	4.55678E-13	65.08
flp-5	C03G5.7	1	3.03286E-08	50.83
flp-6	F07D3.2	1	1.82175E-14	70.86
flp-7	F49E10.3	1	1.98305E-21	90.12
flp-11	K02G10.4	6	5.71409E-13	63.93
flp-13	F33D4.3	2	0.00000345	43.90
flp-14	Y37D8A.15	1	1.86259E-22	91.28
flp-16	F15D4.8	1	2.37294E-13	65.86
flp-18	Y48D7A.2	1	3.86438E-14	70.09
flp-22	F39H2.1	1	2.59659E-14	67.01
flp-34	R09A1.5	1	1.14472E-10	58.54

*Search for homologues of flp-30 and flp-31, which are considered specific to plant-parasitic nematodes (sedentary species) was also performed, however, no matches were found.

Predicted transcripts encoding non-Insulin, non-FLP like peptides

		Pratylenchus penetrans		
**FLPs**	*C. elegans*	# of transcripts	E-value	High bitscore
nlp-1	C01C4.1	2	6.42728E-06	45.82
nlp-2	T24D8.5	1	7.29035E-16	74.71
nlp-10	F37A8.4	1	1.57597E-10	56.23
nlp-12	M01D7.5	1	1.06162E-14	68.55
nlp-13	E03D2.1	2	5.90155E-21	87.81
nlp-15	CC4.2	5	4.24961E-25	99.75
nlp-37	F48B9.4	1	1.36237E-07	48.52
nlp-38	C01A2.7	1	1.99889E-06	45.82
nlp-40	Y74C9A.2	1	1.38484E-09	57.38

Predicted transcript encoding insulin-like peptides

		Pratylenchus penetrans		
**FLPs**	*C. elegans*	# of transcripts	E-value	High bitscore
ins-1	F13B12.5	2	3.08858E-11	59.3066
ins-17	F56F3.6	1	1.13334E-07	49.2914
ins-18	T28B8.2	1	1.23396E-11	58.9214
