# Supplementary material for: The Pratylenchus penetrans Transcriptome as a Source for the Development of Alternative Control Strategies: Mining for Putative Genes Involved in Parasitism and Evaluation of in planta RNAi
Source: PLoS One. 2015 Dec 14;10(12):e0144674. doi: 10.1371/journal.pone.0144674 (PMC4684371; doi:10.1371/journal.pone.0144674)
Supplement: S3 Table — (PDF) [file pone.0144674.s005.pdf]

S3 Table. ***Pratylenchus penetrans*** **transcripts encoding for genes involved in the RNAi pathway, based upon the genes identified for** ***Caenorhabditis elegans*** **[36].** The presence (+) / absence (-) of the corresponding orthologues genes found in the genome of *Pratylenchus coffeae* [13] is also presented.

**Predicted transcripts belonging to *P. penetrans* RNAi pathway**					
			P. penetrans		
	C. elegans	***P. coffeae***	# Transcripts	Best E-value	High bitscore
**Small RNA biosynthetic proteins**	drh-3	+	3	1.48E-85	303.5
	drsh-1	+	2	2.38E-23	94.4
	xpo-1	+	2	0	1018.1
	xpo-2	+	3	4.03E-69	245.4
	dcr-1	+	4	4.84E-64	215.3
	drh-1	+	4	1.38E-29	123.6
	drh-2	-	-		
	pash-1	-	2	8.32E-11	61.6
	rde-4	-	-		
	xpo-3	-	-		
**Amplification proteins**	smg-2	+	7	8.00E-149	456.4
	smg-6	+	3	2.59E-21	98.2
	ego-1	+	8	4.89E-110	364.0
	rrf-2	+	1	3.68E-13	64.7
	rrf-3	+	-		
	rrf-1	+	-		
	smg-5	-	-		
**Spreading proteins**	rsd-2	-	-		
	rsd-3	+	-		
	sid-1	-	-		
	rsd-6	-	-		
	sid-2	-	-		
**Argonautes**	alg-1	+	3	5.25E-76	259.2
	C04F12.1	+	2	1.43E-53	203.0
	alg-4	+	-		
	rde-1	+	-		
	hrde-1	+	-		
	ppw-1	+	-		
	csr-1	+	-		
	ppw-2	+	1	2.26728E-06	45.1
	sago-1	-	-		
	alg-2	+	4	3.13E-72	259.2
	ergo-1	+	-		
	prg-1	+	-		
	hpo-24	+	-		
	nrde-3	+	1	8.16E-13	65.9
	sago-2	+	-		
	T23B3.2	+	-		
	M03D4.7	-	-		
	ZK218.8	-	-		
	prg-2	+	-		
	C06A1.4	-	-		
	C14B1.7	-	-		
	WAGO-1	+	6	2.02E-102	350.1
	WAGO-2	+	2	2.24E-58	211.1
	WAGO-10	-	2	3.10E-28	118.2
	WAGO-4	+	1	1.29E-39	158.3
	WAGO-11	+	7	7.52E-46	175.6
	WAGO-5	+	6	1.40E-45	178.3
**RISC proteins**	tsn-1	-	2	0	915.6
	ain-1	-	1	3.101E-07	53.9
	vig-1	-	-		
	ain-2	-	-		
**RNAi inhibitors**	eri-1	-	1	1.19E-57	198.7
	xrn-2	-	6	7.54E-85	280.8
	adr-2	-	-		
	xrn-1	-	-		
	adr-1	-	-		
	lin-15b	-	-		
	eri-5	-	-		
	eri-6/7	+	-		
	eri-3	-	-		
**Nuclear RNAi effectors**	mut-7	-	1	1.61231E-07	50.8
	cid-1	+	2	1.78E-39	154.8
	ekl-1	+	2	5.79E-14	71.2
	gfl-1	+	1	1.12E-55	182.2
	mes-2	+	2	1.05E-63	220.3
	ekl-4	+	1	1.81E-86	279.6
	mes-6	-	-		
	rha-1	+	6	2.64E-65	221.1
	ekl-6	-	-		
	zfp-1	-	-		
	mut-2	-	-		
	ekl-5	-	-		
	mes-3	-	-		
	mut-16	-	-		
	rde-2	-	-		
