# Supplementary material for: The Pratylenchus penetrans Transcriptome as a Source for the Development of Alternative Control Strategies: Mining for Putative Genes Involved in Parasitism and Evaluation of in planta RNAi
Source: PLoS One. 2015 Dec 14;10(12):e0144674. doi: 10.1371/journal.pone.0144674 (PMC4684371; doi:10.1371/journal.pone.0144674)
Supplement: S4 Table — (PDF) [file pone.0144674.s006.pdf]

S4 Table. List of other carbohydrate active enzymes identified in *Pratylenchus penetrans* transcriptome.

			Best hit to NR database			
**CAZy Families**	**# of transcripts**	**Description of CAZyme**	**min. eValue**	**Bit-Score**	**Hit ACC**	**Top-Hit Species**
**Glycoside Hydrolases**						
GH2	1	Beta-mannosidase	7.45E-150	479.559	ERG82475	*Ascaris suum*
GH13	1	Alpha-amylase	1.94E-57	216.468	ERG85005	*Ascaris suum*
GH18	10	Chitinase domain-containing protein 1	1.57E-116	359.377	ERG84684	*Ascaris suum*
GH20	6	Hexosaminidase d	4.63E-145	450.284	KHN80270	*Toxocara canis*
GH22	1	Destabilase family-containing protein	2.48E-58	195.282	CEF60033	*Strongyloides ratti*
GH25	2	Glycoside hydrolase family 25 protein	1.42E-51	179.874	XP_003290174	*Dictyostelium purpureum*
GH27	3	Alpha-N-acetylgalactosaminidase	2.46E-177	514.998	KHN82558	*Toxocara canis*
GH29	16	Alpha-L-fucosidase	4.17E-78	535.798	CDJ97569	*Haemonchus contortus*
GH31	7	Neutral alpha-glucosidase C	1.89E-110	1156.74	XP_003136312	*Loa loa*
GH32	3	Beta-fructofuranosidase	8.14E-14	77.7962	WP_025417963	*Rhizobium leguminosarum*
GH35	4	Beta-galactosidase	3.20E-135	426.787	KHN82876	*Toxocara canis*
GH37	12	Trehalase	8.12E-130	396.356	AHM26075	*Anisakis simplex*
GH38	10	Glycosyl hydrolase family 38	9.13E-149	441.81	ETN75226	*Necator americanus*
GH47	9	Glycoside hydrolase domain containing protein	1.67E-59	668.307	CDJ83353	*Haemonchus contortus*
GH63	4	Mannosyl oligosaccharide glucosidase	1.94E-179	608.216	KHN75496	*Toxocara canis*
GH84	2	Bifunctional protein ncoat	7.35E-24	645.195	ERG84657	*Ascaris suum*
GH85	1	Cytosolic endo-beta-n-acetylglucosaminidase	4.24E-68	236.113	XP_005597935	*Equus caballus*
**Glycosyl transferases**						
GT1	34	Udp-glucosyltransferase domain containing protein	5.26E-131	407.912	EYC40969	*Ancylostoma ceylanicum*
GT2	6	Beta-mannosyltransferase bre-3	1.76E-174	673.315	ERG82825	*Ascaris suum*
GT3	1	Glycogen synthase	0	1023.85	AAK28335	*Steinernema feltiae*
GT4	9	Alpha-1,3-mannosyltransferase ALG2	3.91E-71	231.106	KHN76171	*Toxocara canis*
GT7	12	Beta-1,4-N-acetylgalactosaminyltransferase bre-4	9.57E-137	409.068	KHN84857	*Toxocara canis*
GT8	10	Glycosyltransferase, family 8	7.67E-88	276.944	ETN68298	*Necator americanus*
GT10	3	Alpha-(1,3)-fucosyltransferase C	1.02E-78	267.7	KHN84318	*Toxocara canis*
GT13	4	Alpha-1,3-mannosyl-glycoprotein 2-beta-N-acetylglucosaminyltransferase	1.16E-100	333.183	ERG81347	*Ascaris suum*
GT14	7	N-acetyllactosaminide β-1,6-N-acetylglucosaminyltransferase	2.25E-65	228.409	KHN76362	*Toxocara canis*
GT16	1	Alpha-1,6-mannosyl-glycoprotein 2-beta-N-acetylglucosaminyltransferase	8.78E-136	421.394	ERG82002	*Ascaris suum*
GT20	4	Trehalose-phosphatase	1.48E-168	504.597	KHN76157	*Toxocara canis*
GT21	1	Ceramide glucosyltransferase	6.66E-153	458.373	ERG80717	*Ascaris suum*
GT22	3	GPI mannosyltransferase 3, partial	5.01E-25	111.694	ETN75500	*Necator americanus*
GT23	5	Alpha 1,6 fucosyltransferase	2.80E-92	301.212	CAI59573	*Caenorhabditis briggsae*
GT24	9	Udp-glucose:glycoprotein glucosyltransferase	9.30E-138	420.239	EYC35440	*Ancylostoma ceylanicum*
GT25	5	Glycosyltransferase 25 family member	1.38E-107	338.576	CEF67199	*Strongyloides ratti*
GT27	14	polypeptide n-acetylgalactosaminyltransferase 3	8.30E-160	725.317	XP_003103194	*Caenorhabditis remanei*
GT31	15	Glycoprotein-n-acetylgalactosamine 3-beta-galactosyltransferase 1	3.11E-172	509.22	ERG84766	*Ascaris suum*
GT33	1	Chitobiosyldiphosphodolichol beta-mannosyltransferase	7.18E-92	297.745	CEF64730	*Strongyloides ratti*
GT35	1	Glycogen phosphorylase	1.87E-46	1434.85	ERG79023	*Ascaris suum*
GT41	3	UDP-N-acetylglucosamine-peptide n-acetylglucosaminyltransferase	1.87E-86	1740.32	CDP93442	*Brugia malayi*
GT43	4	Gactosylgalactosylxylosylprotein 3-beta-glucuronosyltransferase	2.96E-94	296.59	KHN87042	*Toxocara canis*
GT47	7	Exostosin domain containing protein	4.08E-99	311.997	EYB98185	*Ancylostoma ceylanicum*
GT50	2	GPI mannosyltransferase 1	8.96E-38	143.665	CEF64229	*Strongyloides ratti*
GT54	1	alpha- -mannosyl-glycoprotein 4-beta-n-acetylglucosaminyltransferase partial	3.05E-48	182.956	KHN88735	*Toxocara canis*
GT58	4	Dolichyl-P-Man:Man(5)GlcNAc(2)-PP-dolichyl mannosyltransferase	3.29E-82	267.7	KHN72231	*Toxocara canis*
GT65	3	Gdp-fucose protein o-fucosyltransferase 1	4.77E-53	185.267	ERG79773	*Ascaris suum*
GT66	2	Dolichyl-diphosphooligosaccharide--protein glycosyltransferase subunit STT3A	2.05E-38	805.438	KHN82263	*Toxocara canis*
GT68	2	GDP-fucose protein O-fucosyltransferase 2	9.59E-125	392.504	XP_003138384	*Loa loa*
GT76	2	Dolichyl-phosphate-mannose-protein mannosyltransferase	1.26E-38	149.443	XP_003150671	*Loa loa*
**Carbohydrate Esterases**						
CE1	1	S-formylglutathione hydrolase	3.65E-96	298.901	XP_008555559	*Microplitis demolitor*
CE9	1	n-acetylglucosamine-6-phosphate deacetylase	6.98E-18	202.216	CAA77585	*C. elegans*
