# Supplementary material for: The Pratylenchus penetrans Transcriptome as a Source for the Development of Alternative Control Strategies: Mining for Putative Genes Involved in Parasitism and Evaluation of in planta RNAi
Source: PLoS One. 2015 Dec 14;10(12):e0144674. doi: 10.1371/journal.pone.0144674 (PMC4684371; doi:10.1371/journal.pone.0144674)
Supplement: S5 Table — (PDF) [file pone.0144674.s007.pdf]

S5 Table. Significant Blast search results against putative esophageal gland cell secretory proteins of *Meloidogyne incognita* (root-knot nematode) and *Heterodera glycines* (soybean cyst nematode).

P. penetrans transcript	Datbase	Species	Sequence description	E-Value	Bit-Score
Ppen13381_c0_seq1	NEMBASE4	*Meloidogyne incognita*	141302mac02398_1_cluster:_ _esophageal_gland_cell_secretory_protein_2	2.29E-25	68.8822
Ppen15233_c0_seq1	NEMBASE4	*Meloidogyne incognita*	127298hgc00447_1_cluster:_ _esophageal_gland_cell_secretory_protein_26	9.26E-146	166.939
Ppen8995_c0_seq1	NEMBASE4	*Heterodera glycines*	135206hgc11230_1_cluster:heterodera_glycines rep: gland protein g20e03	1.21E-43	143.112
Ppen16443_c0_seq2	NEMBASE4	*Heterodera glycines*	162213mjc04515_1_cluster:heterodera_glycines rep: gland protein g12h04	2.89E-30	132.06
Ppen11950_c0_seq1	NEMBASE4	*Heterodera glycines*	179923pec00345_1_cluster:heterodera_glycines rep: gland-specific protein g4e02	1.28E-40	79.421
Ppen12434_c0_seq2	NEMBASE4	*Heterodera glycines*	128630hgc01884_1_cluster:heterodera_glycines rep: esophageal gland cell secretory protein 6 precursor	2.99E-76	282.407
Ppen12809_c0_seq1	NEMBASE4	*Heterodera glycines*	180010pec00483_1_cluster:heterodera_glycines rep: hypothetical esophageal gland cell secretory protein 12	1.38E-121	246.667
Ppen12536_c0_seq1	EST Nematode_NCBI	*Heterodera glycines*	ck349977hggfha20f07 gland cell ld pcr cdna library heterodera glycines mrna sequence	5.04E-07	54.2195
Ppen11847_c0_seq1	EST Nematode_NCBI	*Heterodera glycines*	ck350205hggfha22h09 gland cell ld pcr cdna library heterodera glycines mrna sequence	3.19E-38	110.359
Ppen11484_c0_seq1	EST Nematode_NCBI	*Heterodera glycines*	ck349337hggfha11h06 gland cell ld pcr cdna library heterodera glycines mrna sequence	4.24E-31	126.616
Ppen11437_c0_seq1	EST Nematode_NCBI	*Heterodera glycines*	ck351745hggfha42g02 gland cell ld pcr cdna library heterodera glycines mrna sequence	3.61E-21	86.7523
Ppen54428_c0_seq1	EST Nematode_NCBI	*Heterodera glycines*	ck349774hggfha17f12 gland cell ld pcr cdna library heterodera glycines mrna sequence	7.43E-12	71.1732
