# Supplementary material for: The Pratylenchus penetrans Transcriptome as a Source for the Development of Alternative Control Strategies: Mining for Putative Genes Involved in Parasitism and Evaluation of in planta RNAi
Source: PLoS One. 2015 Dec 14;10(12):e0144674. doi: 10.1371/journal.pone.0144674 (PMC4684371; doi:10.1371/journal.pone.0144674)
Supplement: S6 Table — (PDF) [file pone.0144674.s008.pdf]

S6 Table. The top best hits of the Blastp search against non-redundant protein database at the NCBI using *P. penetrans* chorismate mutase type 2 domain sequence as query.

Accession	Description	Species	E-value	Query cover	Identi.
CAD29887	Putative chorismate mutase	*Globodera pallida*	7.00E-06	97%	48%
ABR19887	Chorismate mutase precursor protein	*Globodera rostochiensis*	1.00E-05	100%	45%
AEA07499	Chorismate mutase precursor protein	*Globodera pallida*	2.00E-05	100%	45%
AEA07500	Chorismate mutase precursor protein	*Globodera pallida*	2.00E-05	100%	45%
AIE45298	Chorismate mutase precursor	*Globodera ellingtonae*	1.00E-04	100%	44%
WP_013207545	Chorismate mutase	*Ralstonia solanacearum*	3.00E-04	98%	44%
