# Supplementary material for: The Pratylenchus penetrans Transcriptome as a Source for the Development of Alternative Control Strategies: Mining for Putative Genes Involved in Parasitism and Evaluation of in planta RNAi
Source: PLoS One. 2015 Dec 14;10(12):e0144674. doi: 10.1371/journal.pone.0144674 (PMC4684371; doi:10.1371/journal.pone.0144674)
Supplement: S7 Table — (PDF) [file pone.0144674.s009.pdf]

S7 Table. Summary of transcripts encoding proteases with a putative signal peptide and no TMHMM found in *Pratylenchus penetrans* transcriptome.

Peptidase class	Peptidase family	# of transcripts	Best hit Annotation	Best E-value	Best hit Species	Acession
**Aspartic**	A01A	4	ASP-4	0	*Meloidogyne incognita*	ABC88426
**Cysteine**	C01A	15	Cathepsin l-like cysteine proteinase	1.66E-155	*Heterodera glycines*	CAA70693
	C13	2	Prptidase C13	1.04E-149	*Toxocara canis*	KHN76941
	C19	1	Protein djn	1.28E-163	*Ascaris suum*	ERG81404
	C48	3	Sentrin	1.28E-65	*Loa loa*	EJD74295
	C95	2	Phospholipase b-like 2	0	*Caenorhabditis brenneri*	EGT40540
**Metallo**	M10A	4	Matrix metalloproteinase	1.12E-82	*Globodera rostochiensis*	AAR11447
	M12A	6	Protein NAS-isoform b	4.41E-144	*Strongiloides ratti*	CEF59451
	M13A	4	Protein NEP	8.01E-160	*Haemonchus contortus*	CDJ56930
	M14B	1	Carboxypeptidase e	0	*Toxocara canis*	KHN75110
	M38	1	Probable imidazolonepropionase	4.3E-27	*Caenorhabditis brenneri*	EGT51018
**Serine**	S01A	44	Serine protease	2.64E-75	*Heterodera glycines*	CAA74204
	S08B	1	KPC2-type peptidase	0	*Caenorhabditis elegans*	EFP11145
	S09B	1	Dipeptidyl peptidase	8.7E-52	*Toxocara canis*	KHN82489
	S10	3	Serine carboxypeptidase	8.71E-83	*Radopholus similis*	AIC75882
	S28	1	Peptidase s28 domain	3.65E-18	*Ascaris suum*	ERG84535
	S33	5	Epoxide hydrolase	6.89E-100	*Toxocara canis*	KHN84330
